# The Yeast Magmas Ortholog Pam16 Has an Essential Function in Fermentative Growth That Involves Sphingolipid Metabolism

**DOI:** 10.1371/journal.pone.0039428

**Published:** 2012-07-10

**Authors:** Mary K. Short, Joshua P. Hallett, Krisztina Tar, Thomas Dange, Marion Schmidt, Robyn Moir, Ian M. Willis, Paul T. Jubinsky

**Affiliations:** 1 Department of Developmental and Molecular Biology, Albert Einstein College of Medicine, Bronx, New York, United States of America; 2 Department of Biochemistry, Albert Einstein College of Medicine, Bronx, New York, United States of America; 3 Department of Systems and Computational Biology, Albert Einstein College of Medicine, Bronx, New York, United States of America; 4 Department of Pediatrics, Section of Hematology/Oncology, Yale University School of Medicine, New Haven, Connecticut, United States of America; Texas A&M University, United States of America

## Abstract

Magmas is a growth factor responsive gene encoding an essential mitochondrial protein in mammalian cells. Pam16, the Magmas ortholog in *Saccharomyces cerevisiae*, is a component of the presequence translocase-associated motor. A temperature-sensitive allele (*pam16-I61N*) was used to query an array of non-essential gene-deletion strains for synthetic genetic interactions. The *pam16-I61N* mutation at ambient temperature caused synthetic lethal or sick phenotypes with genes involved in lipid metabolism, perixosome synthesis, histone deacetylation and mitochondrial protein import. The gene deletion array was also screened for suppressors of the *pam16-I61N* growth defect to identify compensatory pathways. Five suppressor genes were identified (*SUR4*, *ISC1*, *IPT1*, *SKN1,* and *FEN1*) and all are involved in sphingolipid metabolism. *pam16-I61N* cells cultured in glucose at non-permissive temperatures resulted in rapid growth inhibition and G1 cell cycle arrest, but cell viability was maintained. Altered mitochondria morphology, reduced peroxisome induction in glycerol/ethanol and oleate, and changes in the levels of several sphingolipids including C18 alpha-hydroxy-phytoceramide, were also observed in the temperature sensitive strain. Deletion of *SUR4*, the strongest suppressor, reversed the temperature sensitive fermentative growth defect, the morphological changes and the elevated levels of C18 alpha-hydroxy phytoceramide in *pam16-I61N*. Deletion of the other four suppressor genes had similar effects on C18 alpha-hydroxy-phytoceramide levels and restored proliferation to the *pam16-I61N* strain. In addition, *pam16-I61N* inhibited respiratory growth, likely by reducing cardiolipin, which is essential for mitochondrial function. Our results suggest that the pleiotropic effects caused by impaired Pam16/Magmas function are mediated in part by changes in lipid metabolism.

## Introduction

Mitochondrial associated granulocyte macrophage signaling molecule (Magmas) is a nuclear encoded protein found in the mitochondrial matrix of mammalian cells. It was identified as a transcript that is rapidly induced in the multipotential myeloid cell line PGMD1 by granulocyte-macrophage colony-stimulating factor (GM-CSF) [1]. Magmas is highly conserved [2] and is essential for survival in eukaryotic cells [3], [4], [5]. In addition to the mitochondrial targeting domain [1] a comparison of Magmas homologs from 52 species distributed among animals, plants and fungi revealed three conserved sequence motifs [2]. Kingdom specific amino acid signatures, the potential targeting of the protein to locations other than the mitochondria, and the presence of multiple isoforms in higher eukaryotes suggest that Magmas may function in multiple contexts [2].

Magmas is variably expressed and protein levels are developmentally regulated in mammalian cells [1], [6]. During murine embryogenesis, the highest levels of Magmas are observed in heart, notochord, choroid plexus, cervical ganglion, nasal mucosa and liver. Adult tissues with high Magmas expression include muscle, pancreas, intestinal mucosa, and testes [6].

Magmas expression also differs in normal and neoplastic prostate [7]. The protein is barely detectable in normal prostate glands but increased amounts are observed in some higher-grade tumors. The increased Magmas in malignant cells results from higher amounts of Magmas/cell and not from changes in the number of mitochondria/cell [7]. Investigations in breast cancer correlate increased Magmas expression to poor outcome [8], [9], while studies in pituitary adenoma and Burkitt’s lymphoma cells suggest that increased Magmas levels protect against apoptosis [10], [11]. These data suggest that Magmas has an important role in human cancers.

Human Magmas and its yeast ortholog, Pam 16, are 42% identical and exhibit functional conservation. Expression of human Magmas in yeast fully complements the lethal phenotype of *PAM16* null cells [12], [13] and validates the use of yeast as a model system to study the fundamental properties of the human protein. Pam16 is a regulatory component of the presequence translocase-associated motor (PAM), which is responsible for the transport of proteins into the mitochondrial matrix [14], [15], [16]. Pam16 binding to Pam18 results in recruitment and stabilization of Pam18 to the TIM23 translocase complex, located in the inner mitochondrial membrane. These interactions stimulate the ATPase activity of Ssc1 (the yeast homolog of Hsp70) which produces the driving force responsible for the translocation of proteins into the mitochondrial matrix [12], [13], [16], [17], [18], [19], [20].

To better understand Magmas function we conducted genetic and functional studies on *PAM16*. A temperature sensitive *PAM16* mutation (I61N) was used to identify synthetic sick and lethal (SSL) genetic interactions of *PAM16*. In addition to genetic interactions that support the role of Pam16 in mitochondrial protein import, novel interactions of *PAM16* were identified suggesting its involvement in other functions including lipid metabolism and transcription. Genetic screening also identified gene deletions that suppressed the *pam16*-*I61N ts* proliferation defect. *SUR4*, encoding a fatty acid elongase involved in sphingolipid biosynthesis [21], [22], was the most effective suppressor. Strains containing *pam16-I61N* with or without a deletion of *SUR4* were additionally characterized by proliferation studies, cell cycle analysis, morphology, and sphingolipid profiles. Our results suggest that the deleterious effects of impaired *PAM16*/Magmas activity are mediated in part through changes in sphingolipid metabolites.

## Results

### A Temperature-sensitive *PAM16* Mutant Exhibits Defects in Fermentation and Respiration

Microscopic examination of spores dissected from *PAM16* heterozygous null asci revealed two normal colonies and two single cells (data not shown) demonstrating that haploid null spores are unable to undergo a single cell division and confirming that *PAM16* is essential for proliferation. Thus to characterize the functional roles of Magmas, conditional alleles of *PAM16* were necessary. Temperature sensitive alleles were created by random mutagenesis and screened for their inability to grow at elevated temperatures. Four unique mutant strains that displayed impaired growth at 37°C on glucose and on glycerol/ethanol media (YPGE) contained only a single amino acid substitution. The strain that grew best at 30°C but that did not grow at 37°C had a change at amino acid 61 from isoleucine to asparagine (I61N). This amino acid is identical in Pam16 and Magmas and is located within a sequence of high homology (EX_3_IL) conserved across fungi, plants and animals [2]. *Pam16-I61N* cells incubated for 7 days on YEPD at 37°C remained fully viable and when cultured at 30°C the same numbers of colonies were obtained as on duplicate plates grown at 30°C for 3 days (data not shown).

The temperature dependent growth of the *pam16-I61N* strain on fermentable (glucose) or non-fermentable (glycerol/ethanol) carbon sources is shown in Figure S1. The *pam16-I61N* strain exhibited a strong *ts* phenotype on both media although the temperature required to completely inhibit growth was lower on glycerol/ethanol. Since the *pam16-I61N* mutation affects fermentative growth in addition to respiratory metabolism, it appears that Pam16 may affect metabolic pathways outside of the mitochondrial matrix.

### Synthetic Genetic Interactions

The *pam16-I61N* mutation was introduced into the Y7092 strain (Table 1) to serve as a query strain for SGA analysis. A control strain (Y8835) was screened in parallel and *pam16-I61N xxx*Δ double mutant colonies were identified that failed to grow as well as *pam16-I61N* at 30°C and 32°C. *pam16-I61N xxx*Δ colonies were also incubated at the restrictive temperature of 34°C to identify gene-deletions that suppress the loss of *PAM16* function. Sixty candidate synthetic genetic interactions were initially identified using automated image analysis and visual inspection. Forty-six of these were verified as synthetic sick or lethal (SSL) interactions by random spore analysis (Table 2).

**Table 1:** Saccharomyces cerevisiae strains.

Strain	Genotype	Reference
orfΔarray	*MATa orf*Δ*::KanMX4 his3*Δ *leu2*Δ *met15*Δ *ura3*Δ	[4]
*Y8835*	*MATα can1*Δ*::STE2pr-his5 lyp1*Δ *ura3*Δ*::natR leu2Δ0 his3*Δ*1 met15*Δ*0*	[87]
*Y7092*	*MATα can1*Δ*::STE2pr-his5 lyp1*Δ *ura3*Δ*0 leu2*Δ*0 his3*Δ*1 met15*Δ*0*	[87]
*pam16-I61N*	*MATα pam16-I61N::NatR, can1*Δ*::MFA1-prHIS3 MFα1pr-LEU2 leu2*Δ*0 his3*Δ*1*	This study
*sur4*Δ	*MATα sur4*Δ*:::NatR, can1::MFA1-prHIS3 MFα1pr-LEU2 leu2*Δ*0 his3*Δ*1*	This study
*pam16-I61N sur4*Δ	*MATα pam16-I61N::NatR, sur4*Δ*::URA3, can1 ::MFA1-prHIS3 MFα1pr-LEU2 leu2*Δ*0 his3*Δ*1*	This study
*W303*	*MATa leu2-3, 112 trp1-1 can1-100 ura3-1 ade2-1 his3-11,15*	[85]

**Table 2:** *pam16-I61N* synthetic sick or lethal partners.

Gene name	Systemic name	SSL^a^	petite	Functional group^b^
*CRD1*	ydl142c	+++		Mitochondrial Lipid Metabolism
*ETR1* *	ybr026c	++	X	Mitochondrial Lipid Metabolism
*HFA1* *	ymr207c	+++		Mitochondrial Lipid Metabolism
*LAT1* *	ynl071w	+++		Mitochondrial Lipid Metabolism
*LIP2* *	ylr239c	+	X	Mitochondrial Lipid Metabolism
*LIP5* *	yor196c	+++	X	Mitochondrial Lipid Metabolism
*LPD1* *	yfl018c	+	X	Mitochondrial Lipid Metabolism
*MCT1* *	yor221c	+++	X	Mitochondrial Lipid Metabolism
*PDA1* *	yer178w	+++		Mitochondrial Lipid Metabolism
*PDB1*	ybr221c	++		Mitochondrial Lipid Metabolism
*RPM2*	yml090w^c^	+++	X	Mitochondrial Lipid Metabolism
*TAZ1* *	ypr140w	+		Mitochondrial Lipid Metabolism
*BRE1*	ydl074c	+++		Histone Modification
*DEP1*	yal013w	++	X	Histone Modification
*LGE1*	ypl055c	+++		Histone Modification
*PHO23*	ynl097c	+++		Histone Modification
*RXT2*	ybr095c	++		Histone Modification
*SAP30*	ymr263w	++		Histone Modification
*SIN3*	yol004w	+++	X	Histone Modification
*FMP18*	ykr065c	+++		Mitochondrial Protein Import
*MMM1* *	yll006w	+++	X	Mitochondrial Protein Import
*PHB2*	ygr231c	+++		Mitochondrial Protein Import
*TOM37* *	ymr060c	+++		Mitochondrial Protein Import
*TOM70*	ynl121c	+++		Mitochondrial Protein Import
*YME1*	ypr024w	+++	X	Mitochondrial Protein Import
*PEX29*	ydr479c	+++		Peroxisomal β-oxidation
*PEX30*	ylr324w	+++		Peroxisomal β-oxidation
*PFK2* ?	ymr205c	+		Cytoplasm (Glycolysis)
*ATP11* *	ynl315c	+++	X	Mitochondrial Matrix
*CAF40* *	ynl288w	+++		Cytoplasm
*CDC73* *	ylr418c	+++		Nucleus
*CTK1* *	ykl139w	+++		Cytoplasm And Nucleus
*DBF2* *	ygr092w	+++		Cytoplasm &Bud Neck
*GCR2* *	ynl199c^d^	+++		Nucleus
*KAR3* *	ypr141c	+++		Microtubule, Spindle Pole Body
*MCK1* *	ynl307c	+		Cytoplasm and Nucleus
*MDL2* *	ypl270w	++	X	Mitochondrion Inner Membrane
*MRT1* *	ycr077c	+++		Cytoplasm
*RPL19B* *	ybl027w	+		Cytosolic Large Ribosomal Subunit
*RVS167* *	ydr388w	++	X	Actin Cortical Patch, Cytoplasm
*SWS2* *	ynl081c	+	X	Mitochondrial Small Ribosome
*THR1* *	yhr025w	++		Amino Acid Biosynthesis
*YBR238C*	ybr238c	+++		Mitochondrion
*YDR290W*	ydr290w	+		Unknown
*YJR120W* *	yjr120w	+++		Unknown
*YHL039W*	yhl039w	++		Cytoplasm

**Figure 1:**
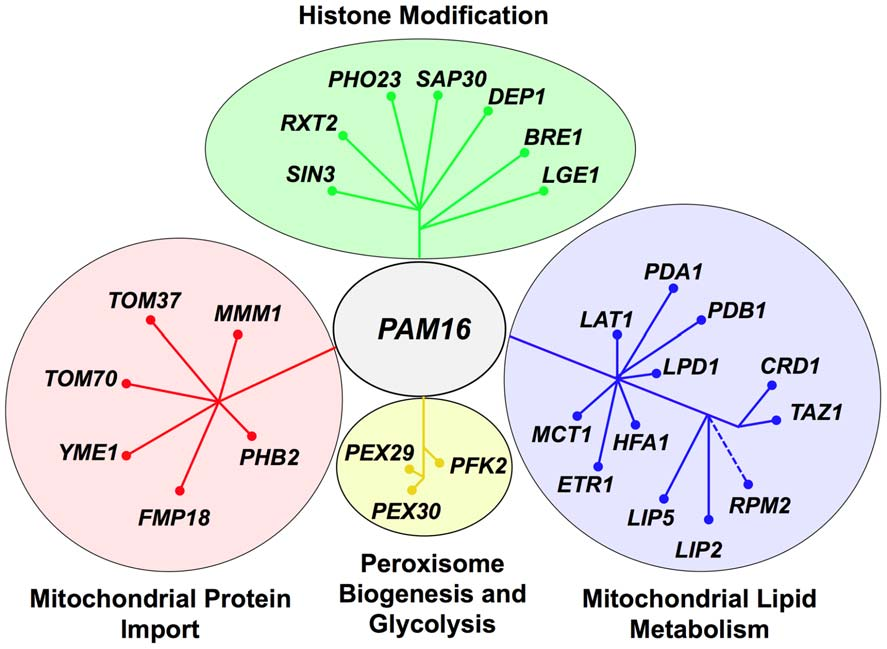
Synthetic lethal partners of *pam16-I61N*. 28 of the 46 SSL partners of *pam16-I61N* were grouped into 4 major biological functions. They are mitochondrial protein import (red), histone modification (green), mitochondrial lipid metabolism (blue), and peroxisome biogenesis and glycolysis (yellow). Genes that are functionally related radiate from a central point.

*: synthetic sick or lethal interaction suppressed by additional deletion of *SUR4*.

a Severity of SSL defect from low+ to high+++.

b Functional group from Figure 1 or GO cellular component or process.

c The interaction with *YML090W,* a dubious open reading frame, was replaced by *RPM2*, an essential gene on the opposite DNA strand whose transcription is inhibited but not eliminated by the *YML090W* deletion (data not shown).

d The interaction with *GRC2* was further confirmed by deletion of *YNL198c,* an overlapping dubious open reading frame on the opposite DNA strand.

Fourteen of the 46 SSL partners had a petite phenotype in the original gene-deletion array but were more severely growth impaired in the double mutant (Table 2). Petite strains do not have respiration competent mitochondria. Since only 227 of the 4285 gene-deletion array strains have a “respiratory growth absent” phenotype [23], the 5.7-fold enrichment for this phenotype in our screen (*p*<0.01) supports the role of *PAM16*/Magmas in specific interactions with genes involved in the regulation of oxidative metabolism [13], [24].

Figure S2 shows an interaction network of all the *PAM16* SSL genes depicted as nodes colored according to gene ontology (GO) biological process designations [25] and edges representing known physical or genetic interactions. When the genes were categorized according to biological function, four major groups were apparent: mitochondrial lipid metabolism, histone modification, mitochondrial protein import, and peroxisome biogenesis and glycolysis (Figure 1). These groups served as the basis for further analysis of Pam16 function.

### Mitochondrial Lipid Metabolism

The functional group containing the largest number of interacting genes established a synthetic relationship between *PAM16* and 12 genes involved in mitochondrial lipid metabolism (Figure 1, blue nodes and Table 2). Ten of these genes encode proteins that are located in the mitochondrial matrix (Table 2) and are involved in mitochondrial specific fatty acid synthesis and the lipoylation of proteins [26], [27], [28]. The two other genes, *CRD1* and *TAZ1,* are essential for the biosynthesis of cardiolipin in the mitochondrial inner membrane. Cardiolipin is important for maintaining mitochondrial membrane potential [29], vacuolar homeostasis [30], [31] and stabilizing respiratory chain complexes [32].

### Histone Modification

The second most numerous SSL gene set includes 7 genes involved in histone modification (Figure 1, green nodes and Table 2). Five of these genes are involved in the Rpd3L histone deacetylase complex, which regulates transcription at distinct promoter regions [33], [34]. Rpd3L negatively regulates genes encoding mitochondrial proteins and proteins involved in meiosis and metabolism. The positively regulated genes are involved in heat shock, osmotic stress and fermentative growth [35], [36]. All the genes in the histone modification group have hundreds of physical and genetic interactions (Saccharomyces Genome Database) besides those shown in the *pam16-I61N* SSL partner set (Figure S2, inner circle). These seven genetic interactions associated with histone modification have not been previously linked to *PAM16*.

### Mitochondrial Protein Import

A third functional cluster consists of genes involved in mitochondrial protein import. Pam16 is a constituent of the presequence translocase-associated motor (PAM) complex. Six genes from the mitochondrial membrane protein import system resulted in significantly impaired growth with *pam16-I61N* (Figure 1, red nodes and Table 2). *MMM1*, *TOM37*, and *TOM70* are mitochondrial outer membrane proteins involved in protein import and additionally have a role in phospholipid biosynthesis and calcium exchange [37], [38], [39]. *PHB2*, *YME1*, and *FMP18* are associated with the mitochondrial inner membrane. Phb2 [40] is a subunit of the prohibitin complex, a chaperone for newly synthesized proteins [41]. *YME1* codes for the catalytic subunit of the mitochondrial inner membrane i-AAA protease complex, which degrades misfolded proteins in the mitochondrial inner membrane and matrix [42], [43]. Of the six genes in this group only Fmp18 (Pam17) has been reported to physically interact with Pam16 [44]. Twenty-two of the 46 SSL partners of *pam16-I61N* were not common to any of the genetic or physical interactions previously identified for the other genes/proteins involved in mitochondrial protein import (data not shown) [23]. This supports our findings that Pam16 is involved in several biological functions.

### Peroxisome Biogenesis and Glycolysis

Negative synthetic interactions were also observed with genes encoding integral membrane proteins of the peroxisome, an organelle responsible for production of fatty acids and energy intermediates during degradation of complex lipids (Figure 1, yellow nodes and Table 2). *PEX29* and *PEX30* are genes required for regulating peroxisome size, number and distribution [45], [46]. *PFK2* encodes phosphofructokinase a major glycolytic enzyme.

### Genetic Suppressors of *pam16-I61N* are Involved in Sphingolipid Metabolism

Five gene deletions (*SUR4*, *ISC1*, *IPT1*, *SKN1,* and *FEN1*) were identified as suppressors of the temperature-sensitive phenotype of *pam16-I61N* at 34°C. All of these genes are involved in sphingolipid metabolism. *SUR4* encodes an elongase that synthesizes very long chain 20–26-carbon fatty acids from C18-CoA primers and is involved in sphingolipid biosynthesis [21]. The Fen1 elongase has a similar role, with a maximum fatty acid chain length of 24 carbons [22]. Isc1 (inositol phosphosphingolipid phospholipase C) hydrolyzes complex phosphorylceramides to produce alpha-hydroxy-phytoceramides and polar headgroups [47], [48]. Deletion of *ISC1* causes defects in mitochondrial function and failure to up-regulate genes involved in aerobic metabolism after the diauxic shift [49], [50].

Ipt1 (inositolphosphotransferase) and Skn1 (a putative glucosyltransferase) are both involved in the synthesis of mannose-(inositol-P)2-ceramide (M(IP)2C), the most abundant complex sphingolipid in yeast [51], [52]. Skn1 and Ipt1 can complement each other in M(IP)_2_C synthesis under limited nutrients whereas *skn1*Δ *ipt1*Δ strains show increased autophagy and DNA fragmentation [53]. Deletion of *FEN1*, *IPT1* or *SKN1* suppressed the 34°C *ts* phenotype of *pam16-I61N* although not as well as deletion of *ISC1* or *SUR4*. Of the five suppressor strains, the *pam16-I61N sur4*Δ strain showed the strongest growth phenotype and was chosen for further analysis. Notably, the suppressor phenotype requires a partially functional *pam16* allele since it cannot suppress the lethality of a *PAM16* deletion (data not shown).

### Synthetic Genetic Interactions of a *pam16-I61N sur4*Δ Double Mutant Query Strain

To understand the functional relationship between *sur4*Δ suppression of *pam16-I61N* and the SSL interactions with *pam16-I61N* we screened the deletion array using a *pam16-I61N sur4*Δ double mutant query strain (Table 2). Nineteen of 46 SSL partners of *pam16-I61N* retained their SSL phenotype while the remaining 27 SSL interactions were suppressed by the deletion of *SUR4*. The *sur4*Δ suppression phenotypes can be divided along functional lines. No SSL interactions in the histone modification group were suppressed. Most SSL interactions with mitochondrial protein import genes were also not suppressed. However, two genes in this group (*TOM37* and *MMM1*) associated with the outer mitochondrial membrane were no longer SSL with *pam16-I61N* when *SUR4* was deleted. Similarly, 9 of 12 SSL interactions of *pam16-I61N* with the mitochondrial lipid metabolism genes were suppressed by deletion of *SUR4*. The genes in this group that remained SSL were *CRD1, PDB1* and *RPM2*.

### Growth Characterization of Wild Type, *pam16-I61N*, *sur4*Δ and the *pam16-I61N sur4*Δ Strains

**Figure 2:**
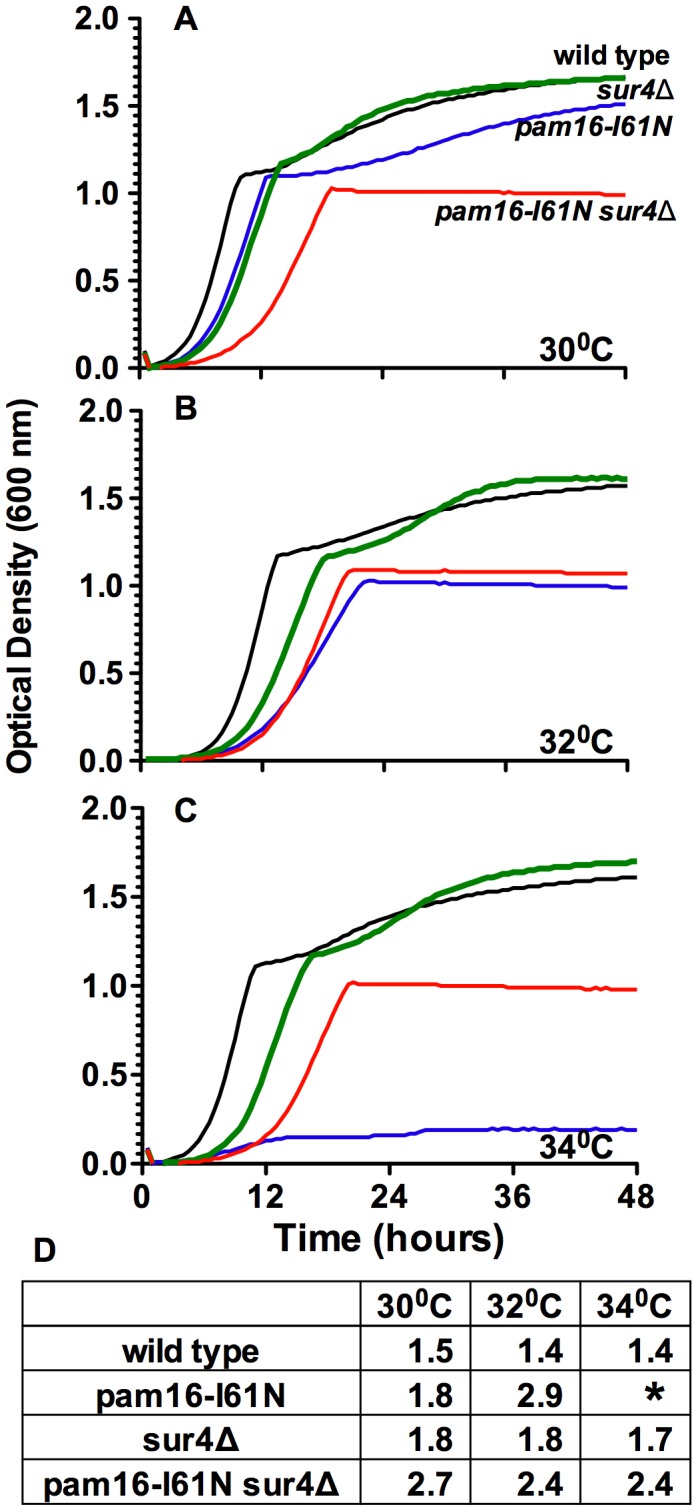
Deletion of *SUR4* suppresses the fermentative growth defect of *pam16-I61N* at 34°C. Effect of temperature on the proliferation of *PAM16* and *SUR4* mutant strains was determined with a Bioscreen incubator. Strains were grown for 48 h at 30°C (A), 32°C (B), or 34°C (C). Growth curves were generated from the mean values of optical density readings taken every 30 minutes from triplicate wells. Wild type (black), *pam16-I61N* (blue), *sur4*Δ (green), and *pam16-I61N sur4*Δ (red). D. Doubling times (h) of yeast strains during log phase fermentative growth. *pam16-I61N never attains log phase growth at 34°C.

The growth of wild type (wt), *pam16-I61N*, *sur4*Δ and *pam16-I61N sur4*Δ strains was evaluated at the three temperatures used in the SGA screens. Growth curves in YEPD media show the typical rapid initial rise in cell density (characteristic of fermentative growth) until the depletion of glucose results in the switch to respiratory metabolism (diauxic shift) and slower growth prior to entry into stationary phase [54], [55] (Figure 2A–C). The fermentative doubling time for the strains at each temperature is shown in Figure 2D. The wt, *pam16-I61N*, *sur4*Δ and the *pam16-I61N sur4*Δ strains all grew at 30°C in glucose although the growth rate of the mutant strains was slower than wt cells.

**Figure 3:**
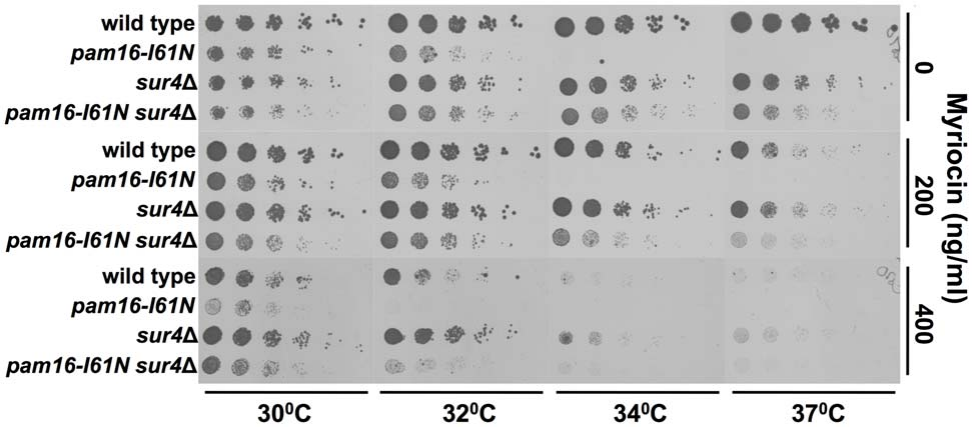
Effects of temperature and disruption of sphingolipid synthesis on proliferation. Five fold serial dilutions of wt, *pam16-I61N*, *sur4*Δ, and *pam16-I61N sur4*Δ were grown on YEPD or YEPD with the indicated concentrations of the serine palmitoyltransferase inhibitor (myriocin) at the temperatures shown. Myriocin (400 ng/ml) impaired the growth of all strains, but neither 200 ng/ml nor 400 ng/ml suppressed the *pam16-I61N (ts)* growth phenotype, unlike the specific suppression by *sur4*Δ.

The reduced ability of *pam16-I61N* to grow on glucose at 32°C and inability to grow at 34°C was partially corrected to wt levels by the deletion of *SUR4* (Figure 2B–D, and Figure 3, top four rows). This data is in agreement with the SGA screen, which showed that proliferation of *pam16I61N* in glucose containing media was restored at 34°C by deletion of *SUR4*. The *sur4*Δ suppression of the *pam16-I61N* growth defect in media containing glucose similarly occurs in *rho^0^* strains, which lack functional mitochondria (Figure S3). The *rho^0^* results suggest that Pam16 provides an essential activity during fermentative growth that is independent of its role in the presequence translocase and other mitochondrial functions.

No growth was observed in *pam16-I61N* after the diauxic shift at 32°C and 34°C (Figure 2B,C; blue line). At 30°C the *pam16-I61N* grew slower than the wt strain and *sur4*Δ strains. In contrast to all the other strains *pam16-I61N sur4*Δ was unable to proliferate at 30°C following the diauxic shift (Figure 2A; red line). This demonstrates that deletion of *SUR4* increases the severity of the respiratory growth defect in the *pam16-I61N* strain.

In addition to very long chain fatty acids, the synthesis of sphingolipids requires a long chain base (LCB) and a polar head group. LCB synthesis is normally required for viability [56] and thus the potential functional relationship between LCBs and *pam16-I61N* was not assessed by screening the deletion array. We therefore examined whether *sur4*Δ suppression of *pam16-I61N* could be phenocopied by pharmacological reduction of LCB synthesis using myriocin, an inhibitor of serine palmitoyltransferase [57]. Myriocin at 400 ng/ml slowed the growth of all strains consistent with the global inhibition of LCB synthesis (Figure 3). However, neither this dose of the inhibitor nor a lower concentration (200 ng/ml, which did not substantially affect growth of the wt strain) suppressed the *pam16-I61N* (*ts*) phenotype.

### Cell Cycle Analysis of Strains Containing *pam16-I61N* and *sur4*Δ

**Figure 4:**
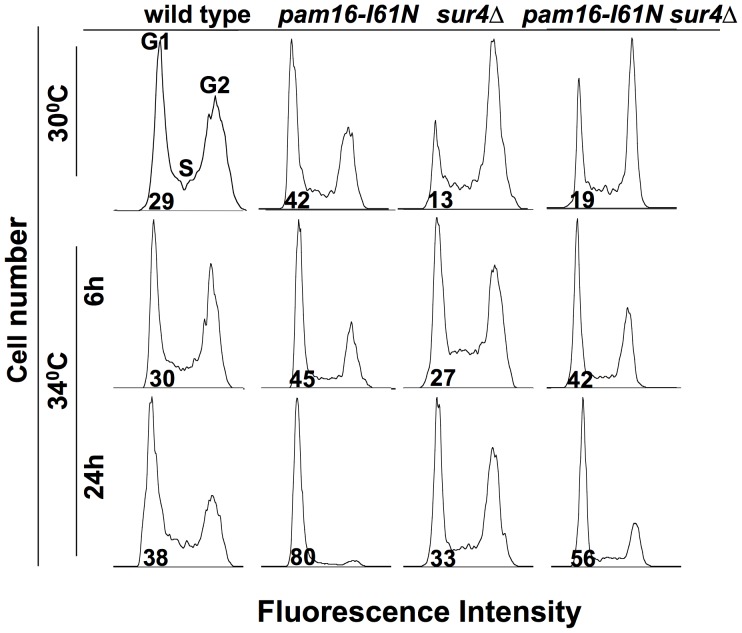
G1 cell cycle arrest of *pam16-I61N* mutant strain at 34°C is relieved by *sur4*Δ. Flow cytometry of wt (Y8835), *pam16-I61N*, *sur4*Δ and *pam16-I61N sur4*Δ. Cultures were kept at mid-logarithmic phase in YEPD at 30°C or shifted to 34°C for 6 or 24 h. Histograms show the number of cells with G1, S and G2 DNA content as measured by Sytox Green fluorescence. The mean percentage of G1 cells is indicated below the G1 peak.

The cell cycle profile of wt, *pam16-I61N, sur4*Δ and *pam16-I61N sur4*Δ strains was assessed by flow cytometry at 30°C and 34°C to determine whether the cells arrested at a specific phase (Figure 4). During normal exponential growth at 30°C in YEPD (Figure 4, row 1), cell cycle analysis showed that 29% of wt cells were in G1 compared to 42% of *pam16-I61N* cells. The *sur4*Δ and the *pam16-I61N sur4*Δ strains had fewer cells in G1 than the wt and *pam16-I61N* strain.

When shifted to the non-permissive temperature of 34°C (Figure 4, row 2) wt cells continued to replicate with a similar cell cycle profile at 6 h and 24 h with slightly more cells accumulating in G1 than at 30°C. In contrast, the majority of *pam16-I61N* cells arrested in G1. This G1 arrest phenotype was quantitatively complete in cells shifted to 37°C (data not shown). The G1 arrest in *pam16-I61N* was relieved in the *pam16-I61N sur4*Δ strain (Figure 4, row 3), and correlates with the improved fermentative growth of the double mutant strain at 34°C (Figure 2C, D, Figure 3).

### Strains Containing *pam16-I61N* and *sur4*Δ have Altered Morphological Features

**Figure 5:**
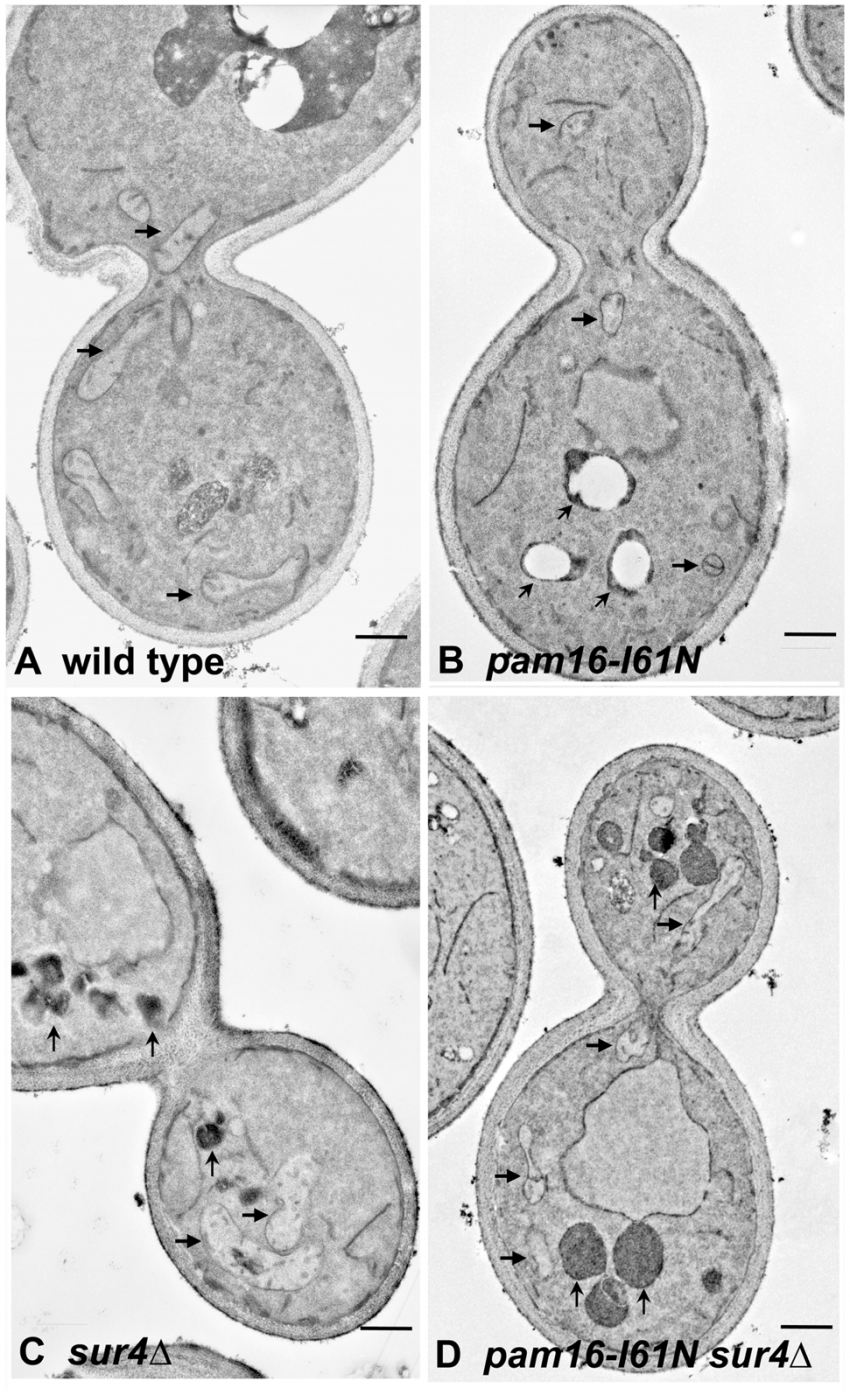
Effects of *pam16-I61N* and *sur4*Δ on yeast morphology. Yeast strains were examined by transmission electron microscopy. *pam16-I61N* (Panel B) had fewer and smaller mitochondria (horizontal arrows) than wt cells (Panel A). *pam16-I61N* cells had vesicles with thickened walls and no electron dense material in the center (upward angled arrows). The *sur4*Δ mutation restores near normal mitochondrial morphology to the *pam16-I61N* strain and increases peroxisome number (upward vertical arrows in Panels C and D)(12,000× magnification; scale bar  = 500 nm).

Wild type, *pam16-I61N*, *sur4*Δ, and *pam16-I61N sur4*Δ cells grown at 32°C were examined by transmission electron microscopy (Figure 5 and Figure S4). Cell walls, cell membranes and the nucleus were similar in all four strains. In contrast, the *pam16-I61N* mutant had fewer and smaller mitochondria than wt cells (Figure 5A, B; horizontal arrows). In addition, the *pam16-I61N* strain contained large vesicles with no electron dense material in the center and thickened walls (small angled arrows). These structures were less apparent in the wt strain.

The mitochondria of the *sur4*Δ strain (Figure 5C) appeared similar to those in the wt strain. The *sur4*Δ strain also contained an additional feature, a large number of highly electron dense vesicles (vertical arrows). *pam16-I61N sur4*Δ cells (Figure 5D) had improved mitochondrial morphology and contained smaller vesicles compared to *pam16-I61N* cells. Similar to *sur4*Δ cells, the *pam16-I61N sur4*Δ strain had numerous other vesicles containing highly electron dense material (Figure 5C, D; upward vertical arrows) that were 10–26 fold more numerous than those present in wt or *pam16-I61N* cells. Live cell fluorescent images (Figure S5) of the strains transformed with a mitochondrial targeted GFP [58] also showed restoration of elongated mitochondrial morphology in *pam16-I61N sur4*Δ compared to the small fragmented mitochondria of the *pam16-I61N* strain.

### Strains Containing *pam16-I61N* and *sur4*Δ have Altered Numbers of Peroxisomes

*Pex29* and *Pex30* are peroxisomal membrane proteins that control peroxisome size, number, and distribution. The absence of either of these genes reduced *pam16-I61N* viability (Figure 1 and Table 2). The electron microscopy of the two strains deleted in *SUR4* showed abundant electron dense vesicles, which could be peroxisomes (Figure 5C, D and Figure S4C, D). To determine if these vesicles were peroxisomes, we performed live cell imaging using a Pot1-GFP fusion protein. Pot1 is specific marker for mature functional peroxisomes.

**Figure 6:**
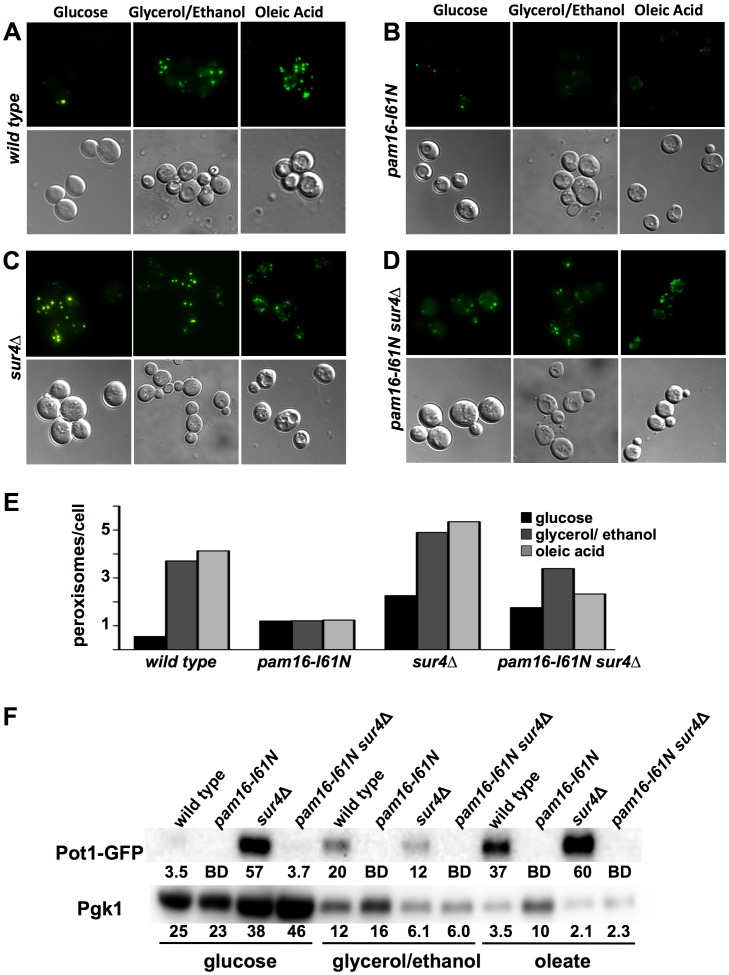
Effects of *pam16-I61N* and *sur4*Δ on peroxisome formation in glucose, glycerol/ethanol and oleic acid containing media. Live-cell fluorescence microscopy (top frames) and the corresponding differential interference contrast (DIC) image (bottom frames) of wt (A), *pam16-I61N* (B), *sur4*Δ (C), and *pam16-I61N sur4*Δ (D) cells containing GFP-tagged Pot1 to identify peroxisomes. Cultures grown in synthetic media at 30°C with glucose were then grown at 32°C for an additional 3 h with glucose, glycerol/ethanol or oleic acid as the carbon source. Quantitation of peroxisomes/cell (E) for each strain grown on the indicated carbon source. Western blot (F) of Pot1-GFP (*M*_r_ = 75 kD) and Pgk1 (*M*_r_ = 45 kD) in wt, *pam16*-*I61N*, *sur4*Δ and *pam16*-*I61N sur4*Δ whole cell lysates (40 µg protein/lane). Pgk1 was used to demonstrate that Pot1 is specifically induced by media with glycerol/ethanol and oleic acid carbon sources. Numbers indicate the quantitation of band volumes for Pot1-GFP (×10^−5^) and Pgk1 (×10^−6^) in pixels. BD indicates that the band was below the level of detection.

Fluorescence microscopy showed that the wt strain grown in glucose had few, small peroxisomes (0.6 peroxisomes/cell). The fluorescence intensity of Pot1-GFP was weak and consistent with the low protein expression observed on western blot (Figure 6A, F). Growing wt cells for 4 h in glycerol/ethanol or oleic acid resulted in an increase in peroxisome number (3.7–4.1 peroxisomes/cell), increased organelle size, and increased fluorescence intensity as expected (Figure 6A and E). These changes were not seen in *pam16-I61N*. The *pam16-I61N* strain had approximately twice as many peroxisomes as wt cells in glucose but exhibited no increase in peroxisome number or fluorescence intensity following culture in glycerol/ethanol or oleic acid (Figure 6B, E).

The *sur4*Δ and the *pam16-I61N sur4*Δ strains had 4.1 and 3.2 fold, respectively, more peroxisomes than the wt strain grown in glucose (Figure 6C–E). In glycerol/ethanol or oleic acid containing media, the number of fluorescent structures increased in *sur4*Δ (2.2–2.4 fold) and the *pam16-I61N sur4*Δ (1.9 fold in glycerol/ethanol, 1.3 fold in oleic acid). The intensity of Pot1-GFP in the *sur4*Δ cells was higher than those in *pam16-I61N sur4*Δ cells. Quantitation of Pot1-GFP levels by western blot confirmed that the two strains with *pam16-I61N* had reduced Pot1 expression (Figure 6F).

Increased peroxisome production in *Saccharomyces cerevisiae* occurs in response to changes in carbon source and involves multiple signaling pathways leading to transcriptional activation of genes encoding peroxisomal proteins (reviewed in [59], [60]). Pot1 expression is repressed in glucose, derepressed in glycerol/ethanol and induced in oleic acid containing media [61]. Although *sur4*Δ and *pam16-I61N sur4*Δ cells grow well in glucose at 32°C (Figure 3), they have a higher baseline peroxisome content (Figure 6) suggesting partial derepression of peroxisomal protein gene transcription. Since *SUR4* deletion results in increased peroxisome content in glycerol/ethanol or oleate these strains have overcome the block in peroxisome induction characteristic of the *pam16-I61N* strain.

### Sphingolipid Composition is Altered in the *pam16-I61N* Strain

Sphingolipids consist of a long-chain base amide linked to a long chain fatty acid. They are critical for plasma membrane structure, lipid raft formation, and play important roles in numerous signaling pathways. The observation that the five suppressors of *pam16-I61N* are involved in sphingolipid metabolism suggested that there could be changes in sphingolipid levels that were biologically important. To investigate this possibility we measured the levels of these molecules in each of the four strains by quantitative mass spectrometry.

Log phase wt, *pam16-I61N, sur4*Δ and *pam16-I61N sur4*Δ strains cultured in YEPD (30°C) were then incubated for 6 h at 34°C, a temperature at which *pam16-I61N* cells lose the ability to proliferate, accumulate in G1, and remain fully viable. *pam16-I61N sur4*Δ cells grow at 34°C but with doubling times that are 1.7× longer than wt cells (Figure 2C, D). The levels of the predominant sphingoid bases, dihydrosphingosine and phytosphingosine, as well as their phosphorylated derivatives were the same for wt and *pam16-I61N* cells (Table S1). The highly abundant C22–26 alpha-hydroxy-phytoceramides and the unsaturated alpha-hydroxy-phytoceramides were also similar in these two strains. The initial steps in *de novo* sphingolipid biosynthesis therefore appear to be unaffected by the *pam16-I61N* mutation.

**Figure 7:**
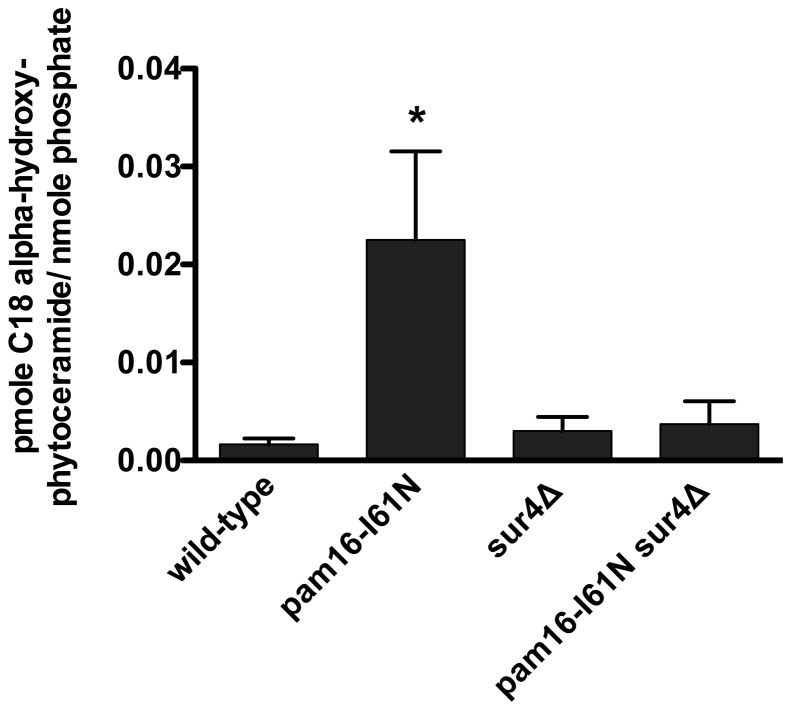
C18 alpha-hydroxy-phytoceramide levels are elevated in the *pam16-I61N strain* compared to wt and are similar to wt in *pam16-I61N sur4*Δ. Overnight yeast cultures of wt, *pam16-I61N*, *sur4*Δ, and *pam16-I61N sur4*Δ strains grown in YEPD at 30°C were diluted into early log phase and grown for 6 h at 34°C. The mean level ± SEM (pmole/nmole phosphate) of C18 alpha-hydroxy-phytoceramide was determined by mass spectrometry. C18 alpha-hydroxy-phytoceramide was 12–15 fold higher in the *pam16-I61N* strain compared to wt, *p = 0.043, n = 3. The *sur4*Δ and *pam16-I61N sur4*Δ strains had C18 alpha-hydroxy-phytoceramide levels that were no different than wt cells.

In contrast, the amounts of several other sphingolipids were different in *pam16-I61N* cells compared to wt cells. C14 and C16 phytoceramide levels were elevated in *pam16-I61N*, while C20–C26 phytoceramides were 3–4 fold less abundant in the *pam16-I61N* strain. This suggests that the reduced levels of long chain phytoceramides were offset by an increase in shorter carbon chain phytoceramides. The levels of alpha-hydroxy-phytoceramides with chain lengths from C16 to C20 were increased in the *pam16-I61N* mutant compared to wt (Table S1 and Figure 7). Most striking was the 14 fold higher levels of C18 alpha-hydroxy-phytoceramide (C18αHP) in the *pam16-I61N* strain compared to wt (p = 0.043) (Figure 7).

Next we compared whether the deletion of *SUR4* affected sphingolipid levels in the *pam16-I61N* strain. *pam16-I61N sur4*Δ resulted in increased levels of many sphingolipids species compared to *pam16-I61N* including the sphingolipid bases dihydrosphingosine and phytosphingosine (Table S1). The phosphorylated forms of these bases were even more elevated than the unphosphorylated forms. *pam16-I61N sur4*Δ also exhibited increases in C20∶1 and C22 alpha-hydroxy-phytoceramide and low levels of the very long chain C24 and C26 molecules as expected with a *SUR4* deletion. Many of these changes in sphingolipids were also observed in the *sur4*Δ strain. Importantly, deletion of *SUR4*, which by itself had no effect on the level of C18 alpha hydroxy phytoceramides, suppressed the elevated levels of this metabolite in the *pam16-I61N* mutant to essentially the wt level (Table S1 and Figure 7). C16αHP levels showed a similar trend in these strains but were not statistically significant.

The dramatic effect of *sur4*Δ in suppressing the 14-fold increase in C18 alpha hydroxy phytoceramides in the *pam16-I61N* strain suggested that a similar effect might underlie the suppressor phenotypes of the other sphingolipid biosynthetic genes. To test this possibility, we constructed suppressor strains by deleting *ISC1*, *IPT1*, *SKN1* and *FEN1* in the *pam16-I61N* strain and performed sphingolipid profiling as before. C18αHP was 7.5 fold higher in the *pam16-I61N* strain compared to wt (Figure S7) and except for *fen1*Δ, the weakest suppressor, the double mutant strains had C18αHP levels similar to wt cells. These data identify C18αHP as an important molecule that may underlie the effects we observe in fermentative growth in the *pam16-I61N* strain.

### Cardiolipin Levels are Decreased in *pam16-I61N* and *pam16-I61N sur4*Δ

Since phospholipids regulate Isc1 activity [62] and *CRD1* and *TAZ1* are SSL with *PAM16* (Figure 1, Figure S2, and Table 2) we measured the major phospholipid content of wt, *pam16-I61N, sur4*Δ and *pam16-I61N sur4*Δ strains by thin layer chromatography (Figure S6). No differences in phosphatidic acid, phosphatidylserine, phosphatidylcholine, phosphatidylethanolamine or phosphatidylinositol were detected suggesting that they did not contribute to the growth and morphology characteristics observed in the *pam16-I61N* strain at non-permissive temperatures. However, cardiolipin levels in *pam16-I61N* were 50% of the wt strain (*p*<0.05) and were further reduced in *pam16-I61N sur4*Δ to 14% of wt (*p*<0.01), n = 4. Since cardiolipin levels were further reduced in *pam16-I61N* by deletion of *SUR4* cardiolipin is not involved in the fermentative growth defect. Decreased cardiolipin levels have been shown to impair oxidative phosphorylation [29] suggesting that the loss of respiratory growth (at all temperatures) in *pam16-I61N sur4*Δ (Figure 2 A–C) may be mediated by the change in cardiolipin levels.

## Discussion

### *PAM16* Genetic Interactions and Function

To identify biological pathways affected by Magmas, conditional mutants were generated in the yeast ortholog *PAM16*. The *pam16-I61N* mutant strain, which was unable to proliferate on glucose at 34°C, was used to identify negative synthetic interactions and to determine the effects of impaired Pam16 function. We found that impaired Pam16 affects growth, cell morphology, mitochondrial protein import, oxidative metabolism, fermentation, lipid/sphingolipid metabolism, peroxisome biosynthesis, and histone modification.

### Suppressors of *pam16-I61N* Participate in Sphingolipid Biosynthesis

**Figure 8:**
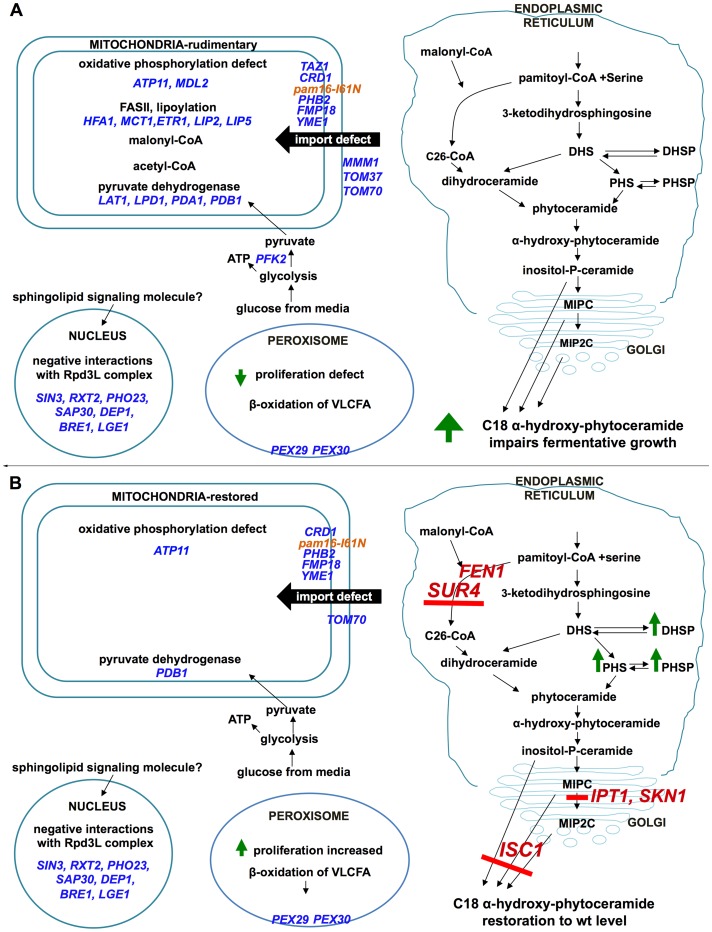
Illustration of the synthetic lethal interactions, metabolic pathways and morphological features of *pam16-I61N* and *pam16-I61N sur4*Δ. (A) *pam16-I61N* strain. Nuclear, mitochondrial, and peroxisome associated negative genetic interactions leading to a SSL phenotype are indicated in blue. These interactions demonstrate the importance of Pam16 in mitochondrial protein import, Rpd3L function in the nucleus, peroxisome biogenesis and energy metabolism. Defects in Pam16 result in rudimentary mitochondria and impaired fermentation and oxidative phosphorylation. The increase in C18αHP likely results from the Isc1 mediated breakdown of complex sphingolipids. The inability of *pam16-I61N* to induce peroxisomes results in decreased beta-oxidation of VLCFA. (B) *pam16-I61N sur4*Δ strain. Negative genetic interactions leading to a SSL phenotype are indicated in blue. Deletion of any one of five genes (red bold font) resulted in a block in the sphingolipid biosynthesis pathway (red bar) suppressing the fermentative growth defect of *pam16-I61N*. The *pam16-I61N sur4*Δ stain has morphologically restored mitochondria, increased numbers of inducible peroxisomes and wt levels of C18 alpha-hydroxy-phytoceramide. Metabolic intermediates and complex sphingolipids synthesized in the endoplasmic reticulum and Golgi are shown in bold font. Green arrows indicate changes up (increased) or down (reduced). Abbreviations: DHS: dihydrosphingosine; DHSP: dihydrosphingosine-1-phosphate; PHS: phytosphingosine; PHSP: phytosphingosine-1-phosphate; VLCFA: very long chain fatty acid.

To detect additional genes functionally connected to *PAM16* we screened the gene-deletion array to find second site suppressors of *pam16-I61N*. Five genes (*SUR4, ISC1, IPT1, SKN1* and *FEN1*) were identified that when deleted rescued the fermentative but not the respiratory proliferation defect of the *pam16-I61N* strain at 34°C. All of these suppressors are involved in sphingolipid biosynthesis (Figure 8B) indicating an important role for sphingolipids in the *pam16-I61N* phenotype [47], [63], [64], [65]. The lack of *pam16-I61N* suppression with myriocin treatment suggests that the reversal of the *pam16-I61N* fermentative growth phenotype by deletion of *SUR4, ISC1, IPT1, SKN1* or *FEN1* is specific.

### *sur4*Δ Rescues a Subset of *pam16-I61N* SSL Interactions

To determine which *pam16-I61N* SSL interactions could be compensated by *sur4*Δ, the gene-deletion array was queried with the double mutant (Table 2). Synthetic lethal genes found in the mitochondrial lipid metabolism and Pfk2, a glycolytic enzyme, were corrected by the absence of *SUR4* demonstrating interdependencies between these pathways (Figure 1 and Table 2). In contrast, deletions of genes involved in the Rpd3L complex and the two *PEX* genes remained synthetic lethal with *pam16-I61N sur4*Δ. Thus, compromised Rpd3L function or compromised peroxisome biogenesis impedes growth in Pam16 deficient cells by mechanisms that are distinct from Pfk2 and the mitochondrial lipid metabolism genes described above.

The deletion of *SUR4* results in increased peroxisome biosynthesis and the restoration of fermentative growth in the *pam16-I61N* strain. However, deletion of *SUR4* is unable to suppress the slow growth phenotype of *pam16-I61N pex29*Δ or *pam16-I61N pex30*Δ. This may reflect a requirement of *PEX29* and *PEX30* for the efficient production of peroxisomes, which cannot be bypassed by deletion of *SUR4*. Increased peroxisome number in *pam16-I61N sur4*Δ cells may be necessary for maintaining energy production via beta-oxidation and/or for reducing detrimental reactive oxygen species that could result from impaired electron transfer in the oxidative phosphorylation complexes [66].

### Biological Features of *pam16-I61N* and *pam16-I61N sur4*Δ

After establishing the importance of the *pam16-I61N* mutation and the *sur4*Δ suppressor we evaluated the biological effects of these two mutations *in vivo*. Significant morphological changes in the *pam16-I61N* strain included small mitochondria and enlarged clear-centered vesicles. Rudimentary mitochondria are a known consequence of defective mitochondrial protein import [67] corroborating the importance of Pam16 in this function. The vesicle accumulation seen in *pam16-I61N* is similar to that described in several mutants strains with defects in endoplasmic reticulum to Golgi trafficking of phospholipids and may reflect altered sphingolipid metabolism in the endoplasmic reticulum [68]. These large vesicles are similar to those that occur during the unfolded protein response [69], [70], [71]. Another feature of *pam16-I61N* cells is their failure to induce peroxisomes when grown on oleate or glycerol/ethanol containing media. This differs from wt strains which increase peroxisome biogenesis and fatty acid oxidation when grown on these carbon sources [72].

The absence of Sur4 reversed many of the biological effects seen in the *pam16-I61N* strain. *pam16-I61N sur4*Δ cells had larger mitochondria, and fewer large clear vesicles than *pam16-I61N.* The improvement in mitochondrial morphology observed with the deletion of *SUR4* in pam16*-I61N* may result from the normalization of Pam16 function or from the reduced activity of specific inhibitory sphingolipids (for example, C18αHP). Significantly, *pam16-I61N sur4*Δ recovered the ability to increase peroxisome number and size when grown in YPGE or oleic acid. The recovery of perixosome synthesis in the double mutant correlates with the resumption of fermentative growth. Alleviation of the unfolded protein response requires lipid biosynthesis in the ER [69] and precursor molecules required for lipid biosynthesis could be supplied by peroxisomal degradation of very long chain fatty acids (VLCFA). Thus recovery from G1 arrest may be due to increased availability of lipid intermediates and of substrates for energy production.

### Elevated C18 Alpha-hydroxy-phytoceramide Levels are Observed with *pam16-I61N*

Sphingolipids are incorporated into plasma and organelle membranes, lipid rafts and cytoplasmic protein complexes. The sphingolipid content of membranes is known to affect many cellular processes including endocytosis, exocytosis and actin cytoskeleton dynamics, calcium signaling, transcription and translation, cell cycle control, differentiation, stress resistance and aging (reviewed in [47], [63]). In addition to their role in membrane biology, sphingolipids and their metabolites can act as intracellular signaling molecules to coordinate nutrition and energy production through sterol metabolism [73], serine utilization, and phospholipid metabolism [74].

The *pam16-I61N* strain had altered levels of many sphingolipid metabolites compared to wt cells (Table S1). Some of the changes in sphingolipid content in *pam16-I61N* cells were corrected towards wt levels by the deletion of *SUR4*. The most dramatic effect occurred for C18αHP, which had 14 fold higher levels in *pam16-I61N* than in wt strains and were restored to wt levels in *pam16-I61N sur4*Δ (Figure 7 and Figure 8A and B). Since the deletion of *SUR4* also restored fermentative proliferation and most of the morphological changes resulting from *pam16-I61N*, C18αHP could be an important mediator of the effects of this mutation. Studies in *Neurospora crassa* also demonstrate the importance of C18αHP. In this organism heat stress and carbohydrate deprivation specifically induced C18αHP, which resulted in cell death [75].

### Potential Mechanisms by which *pam16-I61N* Increases C18αHP Levels During Fermentative Growth

It is unclear whether the dramatic increase in C18αHP levels is a cause or a consequence of the fermentative growth arrest of *pam16-I61N*. However all five suppressor strains reduced the *pam16-I61N* induced elevation of C18αHP (Figure S7) suggesting that increased levels of C18αHP are responsible for the impaired fermentative growth. The elevated levels of C18αHP observed in the *pam16-I61N* strain could result from either increased synthesis or reduced degradation.

Since Pam16 is required for mitochondrial presequence translocase activity and C18αHP is enriched in mitochondrial membranes [50], it is reasonable to consider that *pam16-I61N* would result in increased accumulation of C18αHP by reducing its mitochondrial utilization or degradation. This would require that C18αHP enter the mitochondria through a translocase dependent carrier protein. Alternatively *pam16-I61N* could affect C18αHP degradation pathways instead of its mitochondrial uptake. However neither of these possibilities is consistent with the 5 deletion gene suppressor results.

Instead our data is most consistent with the hypothesis that elevated C18αHP in *pam16-I61N* is predominantly a result of its increased synthesis from complex sphingolipids and not from the *de novo* synthesis pathway (Figure 8). Deletion of *SCS7*, a gene required for *de novo* synthesis of alpha-hydroxy-phytoceramides [76], did not suppress the *pam16-I61N* slow growth phenotype (data not shown). This implies that the accumulation of C18αHP results from increased degradation of complex sphingolipids and the lower levels in suppressed strains results from reduced degradation of complex sphingolipids.

Deletion of *ISC1* results in decreased cellular C18αHP levels [50]. The most abundant Isc1 substrates in wt strains are those sphingolipids with C26 very long chain fatty acids (VLCFA) that are synthesized by Sur4, and the most abundant Isc1 product is C26 alpha-hydroxy-phytoceramide [50]. The *pam16-I61N sur4*Δ strain may have less C18αHP because C26 VLCFAs are not made in *sur4*Δ strains and short chain ceramides are used by the cell in place of ceramides that contain VLCFAs.

Our data suggests that *pam16-I61N* induced elevation of C18αHP is independent of the known mitochondrial activities of Pam16. The growth arrest phenotype of *pam16-I61N* and its reversal in *pam16-I61N sur4*Δ still occurs in the respective *rho^0^* strains lacking functional mitochondria. Pam16 may regulate production of C18αHP by Isc1. The quantitative distribution of Isc1 in microenvironments within the cytoplasm is uncertain although much of its activity occurs in the endoplasmic reticulum during early growth and in the mitochondria after the diauxic shift [49], [50], [77]. Pam16 may regulate Isc1 production of C18αHPs that are typically enriched in the mitochondrial membrane [50]. However the Isc1 preferred substrates, C26 ceramides, are located primarily in the plasma membrane [47]. Neutral sphingomyelinase 2 (nSMase2) the mammalian homolog of Isc1 localizes to the plasma membrane and can functionally replace the yeast gene [78]. It is possible that Isc1 may degrade ceramides at the plasma membrane during fermentative growth. Since Magmas is present in extra mitochondrial locations in the cytoplasm [1], there could be direct interaction between Magmas and SMase2 and their respective counterparts in yeast in the cytoplasm.

### The Inhibitory Effects of *Pam16-I61N* on Respiratory Growth are Independent of its Effect on Fermentative Growth

The Pam16 mutant strain was unable to sustain respiratory growth at the non-permissive temperature (Figure S1 and Figure 2). Unlike fermentative growth, deletion of genes involved in sphingolipid synthesis (*SUR4, ISC1, IPT1, SKN1* and *FEN1*) did not correct the respiratory growth defect (Figure 2 and data not shown). *pam16-I61N* resulted in reduced cardiolipin levels and mitochondrial dysfunction consistent with the severe compromises in mitochondrial morphology (Figure 5 and S5). Decreased levels of cardiolipin have been shown to specifically impair respiratory super complex formation and cytochrome oxidase activity [32], [79], [80].

Studies on Blp the *Drosophila* homolog of Pam16, are consistent with the findings observed in *Saccharomyces cerevisiae* during respiratory growth. Drosophila S2 cells depleted of Blp by RNAi caused mitochondrial membrane depolarization, decreased ATP levels, increased ROS, cell cycle arrest and uncoupling of oxidative phosphorylation [66]. Blp depletion preferentially inhibited cytochrome oxidase activity, which preceded the inhibition of protein import into the mitochondrial matrix. Similarly, a Blp inhibitor (SMMI: small molecule Magmas inhibitor) [81], reduced mitochondrial membrane potential and cytochrome oxidase activity prior to detectable effects on the presequence translocase.

### Magmas in Human Malignancy

Our findings in yeast suggest that Magmas may be involved in human malignancy. Unlike their normal counterparts malignant cells often use aerobic glycolysis for energy requirements. Aerobic glycolysis results in alterations in the levels of many cellular metabolites some of which are involved in the pathogenesis of cancer (reviewed in [82], [83]). Since Pam16 has significant effects on both respiration and fermentation it is likely that Magmas contributes to the metabolic alterations leading to the initiation and maintenance of the malignant phenotype.

## Materials and Methods

### Yeast Strains and Media

Strains used in this study are listed in Table 1. *rho^0^* derivatives were isolated from parental strains by two rounds of growth to saturation in minimal medium containing 2% glucose plus 25 µg/ml ethidium bromide [84], followed by isolation of single colonies on YEPD and testing for no growth on YPGE. Suppressor strains were generated in a *pam16-I61N* mutant background by one-step replacement of each suppressor gene by a hygromycin resistance cassette and selection on YEPD plates with 200 µg/ml hygromycin B (Invitrogen, Carlsbad, CA). Yeast were grown in Yeast Extract and Peptone plus adenine medium with 2% glucose (YEPD) or 2% glycerol and ethanol (YPGE) on agar plates or in liquid culture. Synthetic genetic array experiments used yeast nitrogen base (Becton, Dickinson and Co. Sparks, MD) with added specific amino acid drop out selection and nourseothricin (cloNAT; WERNER BioAgents, Germany), canavanine, thialysine, and G418 (Sigma, St. Louis, MO) for serial selection procedures. In the experiments involving peroxisome analysis the growth medium was yeast nitrogen base plus amino acids and 2% glucose, glycerol and ethanol, or 0.25% oleic acid and 0.3% Brij35. YEPD supplemented with myriocin (Sigma) at the indicated concentrations was used in the serine palmitoyltransferase inhibition experiments.

### *PAM16* Mutations

*PAM16 ts* mutants were made by error-prone PCR using yeast strain W303 [85]. *PAM16* cDNA encoding amino acids S41 through D142 was subcloned into pCR-TOPO (Invitrogen) and used as the template for mutagenesis. The error-prone PCR products were subcloned into wt *PAM16* in pRS315 and transformed into *E. coli* XL-1B (Agilent, Santa Clara, CA). Bacterial colonies were scraped from plates and plasmid DNA was extracted. W303a haploid yeast containing *pam16*Δ*::HIS3* and a wt copy of *PAM16* on pRS316 was transformed with the mutant plasmid library. After eviction of the plasmid by growth at 25°C on YEPD/FOA [86], surviving colonies were plated on YEPD or YPGE at 18°C, 30°C, and 37°C to identify *ts* conditional mutants. The plasmid DNA was sequenced to determine the mutation responsible for the conditional phenotype.

### Synthetic Genetic Array (SGA)

Query strains were derived from Y7092 (Table 1) with a nourseothricin resistant gene located adjacent to the chromosomal *pam16-I61N* mutant or replacing non-essential genes. The *pam16-I61N sur4*Δ double mutant was derived from the *pam16-I61N* query by replacing the *SUR4* coding region with *URA3*. The array of deletion strains has been described by Tong and Boone [87]. Automated construction of double mutants was achieved by crossing the set of viable gene-deletion mutants with the chosen query strain. Deletion strains were arrayed on 7 plates at a density of 1536 colonies per plate with duplicates of each gene-deletion. Each SGA screen was performed twice using a Singer RoToR HDA robot (Singer Instruments, Somerset, UK) for all pinning procedures. Plates were photographed 30 h after the final pinning step. The growth of double mutant colonies obtained using a control strain (Y8835) or the *PAM16* mutant strain (*pam16-I61N*) were compared by visual inspection and by computer-based image analysis (ColonyImager software) [88]. All synthetic genetic interactions were validated by random spore analysis [89].

### Proliferation Assay

Log phase cultures of selected strains were counted by hemocytometer, diluted to 5×10^5^ cells/ml, and grown in YEPD in a microtiter plate shaken at high speed at the indicated temperature for 48 h in a Bioscreen C MBR (Growth Curves USA, Piscataway, NJ). Growth curves were derived from OD 600 nm readings of triplicate culture samples measured every 30 minutes. Standard deviations were less than 0.1% of each mean value. Doubling times were calculated from the slope of the natural log OD 600 nm vs. time at cell concentrations between OD 0.2–0.6. Two independent experiments were performed.

### Flow Cytometry

Cell cycle and cell size were analyzed by fluorescence activated cell sorting on a LSRII analyzer (Becton Dickinson, Franklin Lakes, NJ) at the Albert Einstein College of Medicine Flow Cytometry Core Facility. Log phase cultures of yeast strains grown in YEPD at 30°C were switched to 34°C for the times indicated, washed in buffer and fixed in 70% ethanol/H_2_O. Fixed cells were digested with RNAse A and proteinase K (Sigma) and stained with Sytox green (Invitrogen). Cells were sonicated for 5 seconds at 30% power with a Sonic Dismembrator Model 500 (GE Healthcare, Piscataway, NJ) prior to analysis. Quantitation of percent cells in cell cycle stages was determined using FloJo analysis software (BD Biosciences, San Jose, CA). Data shown is representative of 3 independent experiments.

### Electron Microscopy

Cultures of wt, *pam16*-*I61N*, *sur4*Δ and *pam16*-*I61N sur4*Δ grown overnight in YEPD at 30°C were split into prewarmed 32°C YEPD and grown for 4–6 h until OD 600 nm reached 0.500. 9.5 ml of each culture was fixed (2.5% glutaraldehyde in 0.1 M sodium cacodylate buffer) and postfixed with 1% osmium tetroxide, followed by 2% uranyl acetate, dehydrated through a graded series of ethanol and embedded in LX112 resin (LADD Research Industries, Burlington, VT). Sections were cut on a Reichert Ultracut UCT, stained with uranyl acetate followed by lead citrate and viewed on a JEOL 1200EX transmission electron microscope at 80 kv at the Analytic Imaging Facility at the Albert Einstein College of Medicine (AIF). At least 100 cells of each strain were examined. Mitochondria and peroxisomal number and cell and mitochondrial cross sectional areas were measured for every cell contained in two low power fields for each strain using ImageJ 1.40 g software (National Institutes of Health, USA). Differences between strains were determined by comparing the total organelle area divided by total cell area. Two independent experiments were performed.

### Live Cell Fluorescence Microscopy

Yeast strains carrying C-terminal GFP-tagged Pot1 to identify peroxisomes were grown overnight in synthetic media supplemented with 2% dextrose. After dilution to an OD 600 nm of ∼0.3 (to obtain log phase cultures) in synthetic media supplemented with 2% dextrose, 2% glycerol and ethanol, or with 0.25% oleic acid and 0.3% Brig 35 (Sigma), cultures were grown for an additional 3 h at 32°C. Yeast strains with a plasmid encoding a mitochondrial leader sequence-GFP fusion protein (pYX232-mtgfp) [58] were grown overnight in YEPD at 30°C, split into prewarmed 32°C YEPD and grown for 4 h. Cells were harvested prior to live-cell fluorescence microscopy by centrifugation at 3000 rpm for 5 minutes and resuspended in their respective media. Live-cell fluorescence of the strains was monitored using a fluorescence microscope (Olympus BX61) at the AIF with a 60× numerical aperture 1.4× objective (PlanApo). Fluorescence or differential interference contrast (DIC) images were captured with a cooled CCD camera (Cooke Sensicam QE, Cooke Corporation, Romulus, MI) using IPLab 4.0 software (BD Biosciences). Images were analyzed with ImageJ software 1.42q (National Institutes of Health, USA). In the Pot1-GFP experiments the number of peroxisomes per cell was the average obtained from at least 100 cells. Two independent experiments were performed.

### Western Blot

Forty micrograms of total cellular protein from each yeast strain was solubilized in Laemmli sample buffer and the proteins were separated by SDS-PAGE. The proteins were transferred to a nylon membrane (Westran S, Whatman Inc., Sanford ME) in a TRIS/glycine-20% methanol solution (100 volt-hour) at 14°C. Membranes were rinsed, blocked, and then incubated with a monoclonal antibody to GFP (Roche Applied Science, Indianapolis, IN). After washing, the membrane was immersed for 1 h with peroxidase-labeled secondary antibody, washed four times with buffer containing 50 mM TRIS at pH 7.5/150 mM NaCl/0.1% Tween 20 (TBST), for 15 minutes each, and developed in luminol substrate (Immobilon, Millipore, Billerica, MA). The resulting image was captured with the ImageQuant LAS4000 biomolecular imager and the signal intensities of specific bands determined using ImageQuant TL7.0 software (both from GE Healthcare Life Sciences, Piscataway, NJ) and expressed as band volumes. Blots were stripped and reprobed with a monoclonal antibody to yeast Pgk1 (Invitrogen) Pgk1 is a protein induced by glucose [90].

### Sphingolipid Analysis

Yeast strains were grown in liquid culture in YEPD overnight at 30°C, diluted to 1×10^6^ cells/ml, and incubated for 6 h at 34°C in a shaking water bath. Cultures were washed twice in cold water and snap frozen in liquid nitrogen. Lipids were quantitatively analyzed at the Lipidomics Analytical Unit of the Medical University of South Carolina (http://www.musc.edu/BCMB/lipidomics/index.shtml) using multicomponent LC/MS analysis or simultaneous ESI/MS/MS analysis on a Thermo Finnigan TSQ 7000 triple quadrupole mass spectrometer operating in a multiple reaction monitoring (MRM) positive ionization mode as described [91]. The quantity of each sphingolipid was normalized to organic phosphate determination. The results shown in Table S1 and Figure 7 are the mean values from three separate experiments. Sphingolipid content is expressed as pmole sphingolipid/nmole phosphate. Values were compared using a one-way ANOVA followed by Dunnett’s multiple comparison test.

### Phospholipid Analysis

Relative phospholipid levels were determined according to the method of Vaden et al. [92]. Yeast strains were grown in YEPD at 32°C containing 10 µCi ^32^P*_i_* ml^−1^ at an OD 600 nm of 0.025 and incubated to an OD of 0.800. Cells were washed in H_2_O once and digested with zymolase to form spheroplasts. Phospholipids were extracted from the spheroplasts and dissolved in 2∶1 chloroform/methanol. Samples were applied to Whatman LK5 silica gel TLC plates (GE Healthcare) and separated in chloroform/ethanol/water/triethylamine 30/35/7/35 v/v for 2 h, dried, and run again in the same solvent system for another 2 h. The signal intensity of each band was captured on a phosphoimager screen, and was analyzed using a Fujifilm FLA-7000 Phosphoimager and ImageQuant software (GE Healthcare). Four independent experiments were performed.

## Supporting Information

Figure S1 **Growth characteristics of yeast strains.** Five fold serial dilutions of the wt query strain, and *pam16-I61N*, were spotted on YEPD or YPGE plates. *pam16-I61N* displayed temperature sensitive growth inhibition on medium containing glucose or glycerol/ethanol at 32°C and growth arrest at 34°C and 37°C.
(TIF)

Figure S2 **Synthetic lethal partners of** ***Pam16-I61N*** **.** Schematic of genetic network interactions of SSL partners of *Pam-I61N*. 46 deletion strains were synthetic sick or lethal when paired with mutant Pam16. The synthetic lethal partners were assigned by the Osprey software program [93] into 10 of 17 broad gene ontology (GO) process categories; including 20 in Cell Organization and Biogenesis (purple), 14 in Metabolism (blue), 2 in Unknown Function (gray), 2 in Protein Transport (hot pink), and 1 each in the Stress Response (flesh), Cell Cycle (green), DNA Metabolism (beige), Protein Amino Acid Phosphorylation (brown), and Transcription (turquoise). The schematic shows the genes with their GO category as a colored circle with the lines connecting them designating a physical or genetic interaction. The genes were arranged alphabetically in two concentric circles with the inner circle containing the genes with the most interactions with other members of the set and those in the outer circle having fewer interactions. Edge lines between nodes are colored to represent the experimental system used to determine the association. These are synthetic lethality (light green), affinity capture (navy blue), dosage rescue (orange), two-hybrid (aqua) phenotypic enhancement (pea green), reconstituted complex (rust), biochemical activity (dark blue). Two genes, *YNL198c* and *YML090W*, were open reading frames in the SGD. *YNL198c* was deleted from our SSL set as redundant because it overlaps the opposite DNA strand coding for *GCR2*. *YML090W* was replaced by *RPM2*, an essential gene on the opposite DNA strand whose transcription is inhibited but not destroyed by the *YML090W* deletion (Table 2).
(TIF)

Figure S3 **Strains lacking functional mitochondria** **have the same temperature sensitive effects on growth as their parental strains.** Five fold serial dilutions of two independent *rho^0^* strains derived from wt, *pam16-I61N*, *sur4*Δ, and *pam16-I61N sur4*Δ were spotted on YEPD plates at the temperatures indicated. *pam16-I61N* temperature sensitive growth inhibition at 34°C and 37°C and suppression in *pam16-I61N sur4*Δ occurred in cells lacking functional mitochondria suggesting that these effects may be mediated elsewhere in the cell. n = 2.
(TIF)

Figure S4 **Effects of** ***pam16-I61N*** **and** ***sur4*** **Δ on yeast morphology.** Transmission electron microscopy of yeast strains wt (A), *pam16-I61N* mutant strain (B), *sur4*Δ (C), and *pam16-I61N sur4*Δ (D) are shown in a low power field to demonstrate that the cells depicted in Figure 5 are representative of the entire population. (5,000× magnification; bar = 1 um).
(TIF)

Figure S5 **Effects of** ***pam16-I61N*** **and** ***sur4*** **Δ on mitochondrial morphology.** Live-cell fluorescence microscopy of wt (A), *pam16-I61N* (B), *sur4*Δ (C) and *pam16-I61N sur4*Δ (D) containing mitochondrial leader-GFP to display mitochondrial morphology at the semi-permissive temperature (32°C) (left panels) and the corresponding differential interference contrast image are shown in the right panels. The fragmented mitochondria morphology observed in *pam16-I61N* was restored to the wt elongated reticular mitochondrial morphology in *pam16-I61N sur4*Δ.
(TIF)

Figure S6 **Steady state phospholipid levels in wt,** ***pam16-I61N*** **,** ***sur4*** **Δ,** ***pam16-I61N sur4*** **Δ and** ***crd1*** **Δ.** Phospholipids from wt, *pam16-I61N*, *sur4*Δ, *pam16-I61N sur4*Δ and *crd1*Δ strains were analyzed by thin layer chromatography. (A) Phosphoimage of a representative plate showing the separation of ^32^P orthophosphate labeled organic extracts from the indicated strains. **†** Phospholipids at low levels in *sur4*Δ may be CDP-DAG and LPI/LPC. (B) Quantitation of phospholipids from each strain expressed as % total (cpm specific phospholipid/cpm total phospholipid in sample). Values shown are mean ± SEM. Only cardiolipin levels were significantly different. Wt *vs pam16-I61N*, *p<0.05; wt *vs pam16-I61N sur4*Δ, ** *p*<0.01. Student’s unpaired two-tail t-test. (n = 4). Quantitation of total phospholipid (PL) cpm per lane is shown in the lower right panel. Since wt and *pam16-I61N* have similar amounts of monovalent phospholipids, these phospholipids were not responsible for the slow growth phenotype and morphological changes occurring in *pam16-I61N*. *pam16-I61N* had lower levels of cardiolipin (CL) than wt, and *pam16-I61N sur4*Δ had even more reduced levels. Therefore CL levels do not account for the suppression of the *pam16-I61N* fermentative growth defect by deletion of *SUR4* but could be involved in the respiratory growth defect (Figure 2). Abbreviations: PDME, phosphatidyldimethylethanolamine; PA, phosphatidic acid; PE, phosphatidylethanolamine; PS, phosphatidylserine; PI, phosphatidylinositol; PC, phosphatidylcholine; LPI/LPC, lysophosphatidylinositol or lysophosphatidic acid; CDP-DAG, cytidine diphosphate diacylglycerol.
(TIF)

Figure S7 **Elevated C18 alpha-hydroxy-phytoceramide levels in** ***pam16-I61N are*** **reduced in** ***pam16-I61N*** **double mutants that suppressed the** ***ts*** **slow growth phenotype.** The indicated strains were grown to early log phase in YEPD at 30°C before shifting the temperature to 34°C. Cells were harvested after 6 h at the elevated temperature. The normalized mean level ± SEM of C18 alpha-hydroxy-phytoceramide was determined by mass spectrometry (n = 4). Wt (black), *pam16-I61N*, (blue), *pam16-I61N sur4*Δ (red), *pam16-I61N isc1*Δ (yellow), *pam16-I61N ipt1*Δ (green), *pam16-I61N skn1*Δ (purple), *pam16-I61N fen*Δ (orange). C18 alpha-hydroxy-phytoceramide was 7.5 fold higher in the *pam16-I61N* strain compared to wt in these experiments. Except for *fen1*Δ, the weakest suppressor, the double mutant strains had C18 alpha-hydroxy-phytoceramide levels similar to wt.
(TIF)

Table S1 **Comparison of sphingolipid content in wild type,** ***pam16-I61N, sur4*** **Δ** ***and pam16-I61N sur4*** **Δ strains.** The sphingolipid content of strains growing in logarithmic phase at 34°C were measured by mass spectrometry. The quantity of each sphingolipid was normalized to organic phosphate. The values shown are the means ± SEM (pmole sphingolipid/nmole phosphate). n = 3. Abbreviations: αOH, alpha-hydroxy; Cer, ceramide; dh, dihydro; Sph, sphingosine; P, phosphate.
(XLSX)
