# Supplementary figures and images for: The Yeast Magmas Ortholog Pam16 Has an Essential Function in Fermentative Growth That Involves Sphingolipid Metabolism
Source: PLoS One. 2012 Jul 10;7(7):e39428. doi: 10.1371/journal.pone.0039428 (PMC3393719; doi:10.1371/journal.pone.0039428)

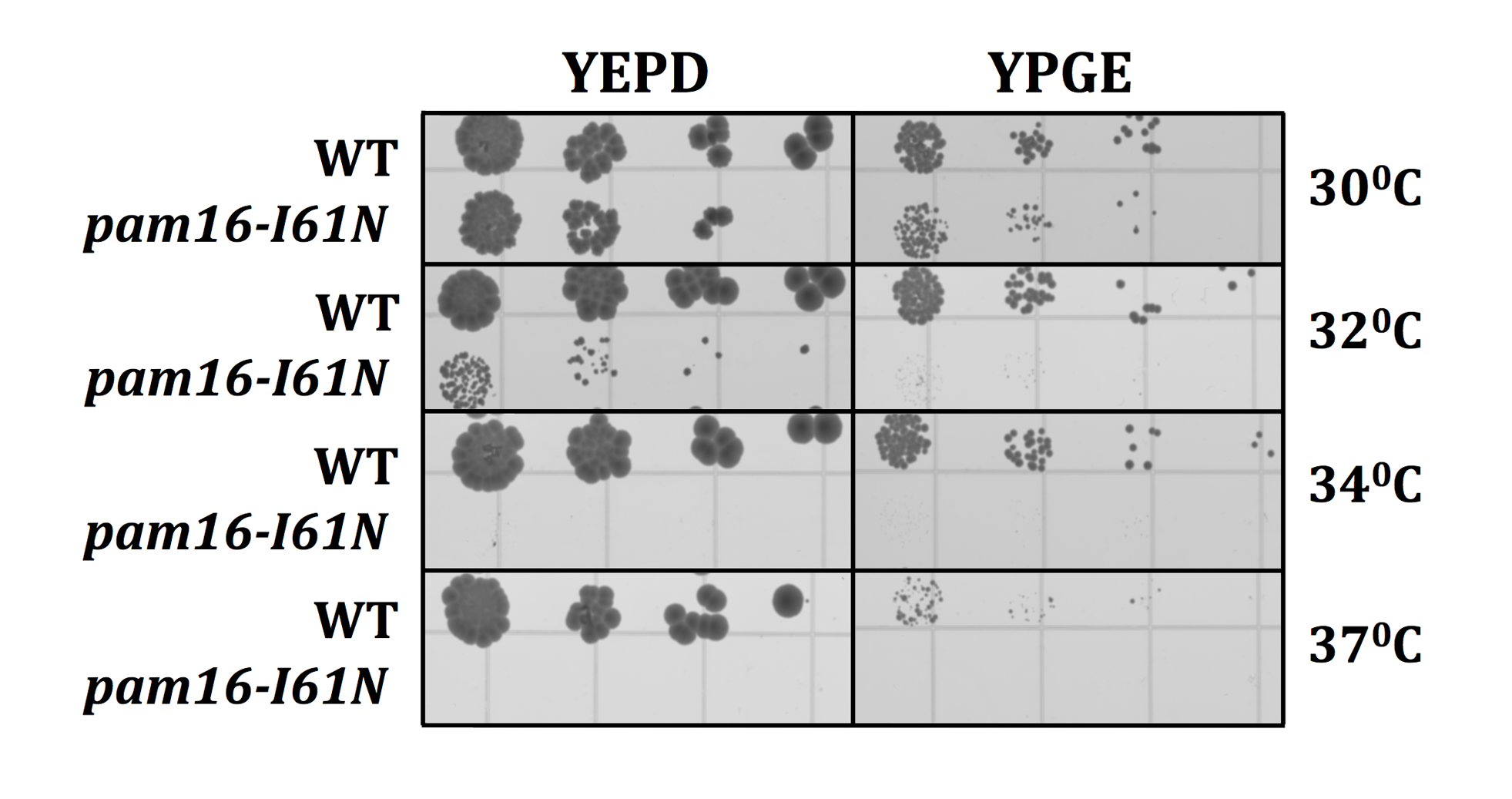

Supplement: Figure S1 — Growth characteristics of yeast strains. Five fold serial dilutions of the wt query strain, and pam16-I61N, were spotted on YEPD or YPGE plates. pam16-I61N displayed temperature sensitive growth inhibition on medium containing glucose or glycerol/ethanol at 32°C and growth arrest at 34°C and 37°C. (TIF) [file pone.0039428.s001.tif]

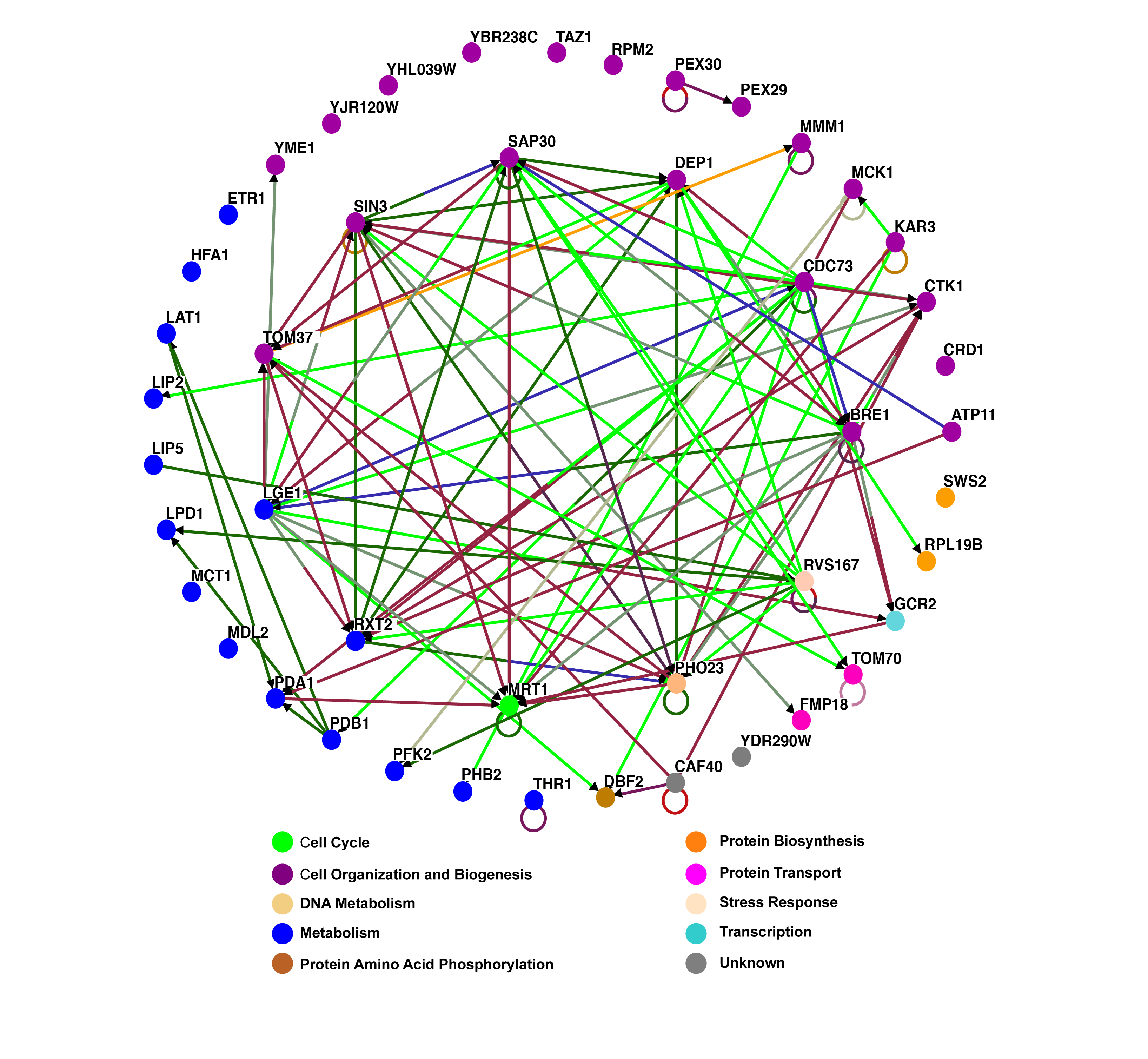

Supplement: Figure S2 — Synthetic lethal partners of Pam16-I61N . Schematic of genetic network interactions of SSL partners of Pam-I61N. 46 deletion strains were synthetic sick or lethal when paired with mutant Pam16. The synthetic lethal partners were assigned by the Osprey software program [93] into 10 of 17 broad gene ontology (GO) process categories; including 20 in Cell Organization and Biogenesis (purple), 14 in Metabolism (blue), 2 in Unknown Function (gray), 2 in Protein Transport (hot pink), and 1 each in the Stress Response (flesh), Cell Cycle (green), DNA Metabolism (beige), Protein Amino Acid Phosphorylation (brown), and Transcription (turquoise). The schematic shows the genes with their GO category as a colored circle with the lines connecting them designating a physical or genetic interaction. The genes were arranged alphabetically in two concentric circles with the inner circle containing the genes with the most interactions with other members of the set and those in the outer circle having fewer interactions. Edge lines between nodes are colored to represent the experimental system used to determine the association. These are synthetic lethality (light green), affinity capture (navy blue), dosage rescue (orange), two-hybrid (aqua) phenotypic enhancement (pea green), reconstituted complex (rust), biochemical activity (dark blue). Two genes, YNL198c and YML090W, were open reading frames in the SGD. YNL198c was deleted from our SSL set as redundant because it overlaps the opposite DNA strand coding for GCR2. YML090W was replaced by RPM2, an essential gene on the opposite DNA strand whose transcription is inhibited but not destroyed by the YML090W deletion (Table 2). (TIF) [file pone.0039428.s002.tif]

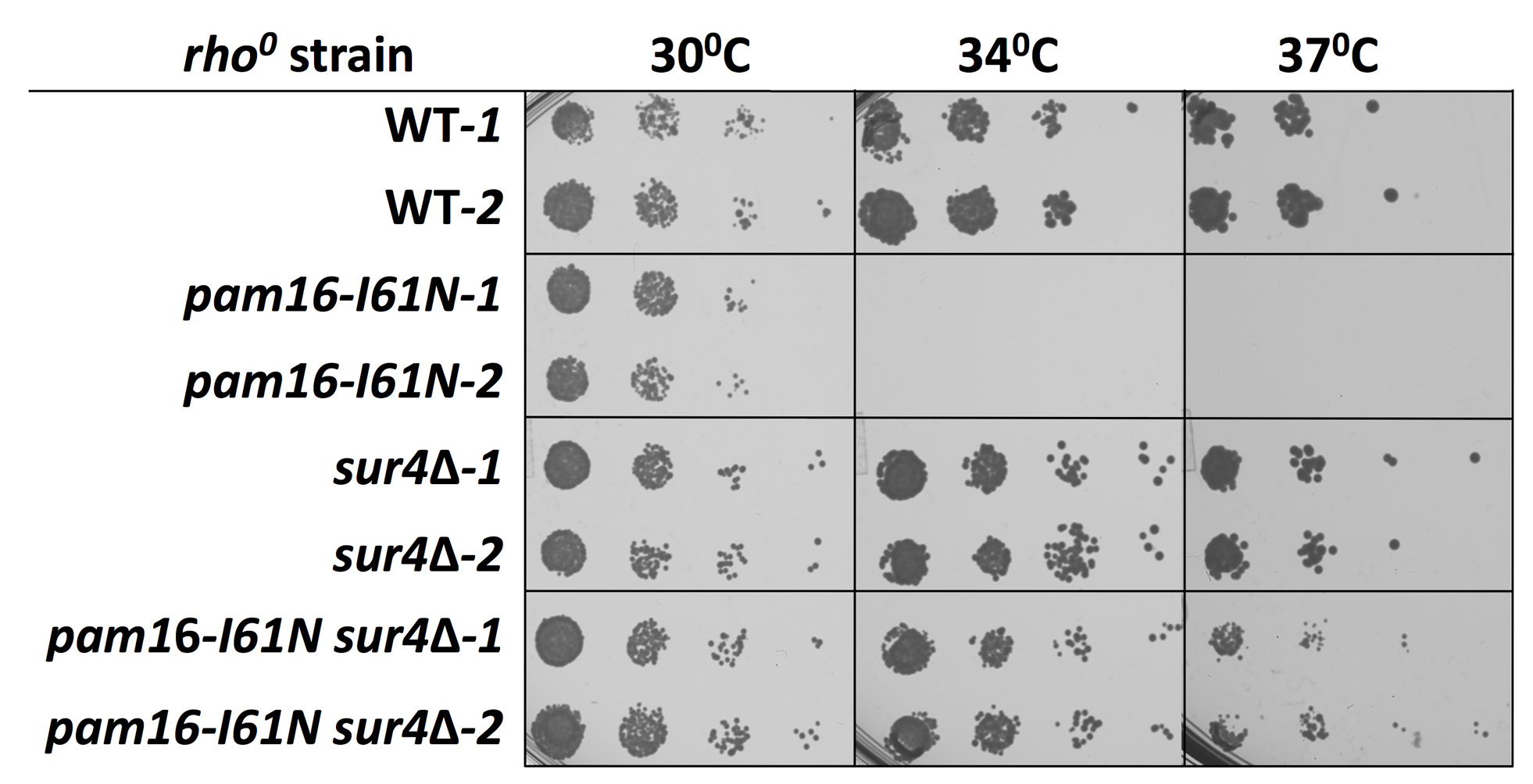

Supplement: Figure S3 — Strains lacking functional mitochondria have the same temperature sensitive effects on growth as their parental strains. Five fold serial dilutions of two independent rho0 strains derived from wt, pam16-I61N, sur4Δ, and pam16-I61N sur4Δ were spotted on YEPD plates at the temperatures indicated. pam16-I61N temperature sensitive growth inhibition at 34°C and 37°C and suppression in pam16-I61N sur4Δ occurred in cells lacking functional mitochondria suggesting that these effects may be mediated elsewhere in the cell. n = 2. (TIF) [file pone.0039428.s003.tif]

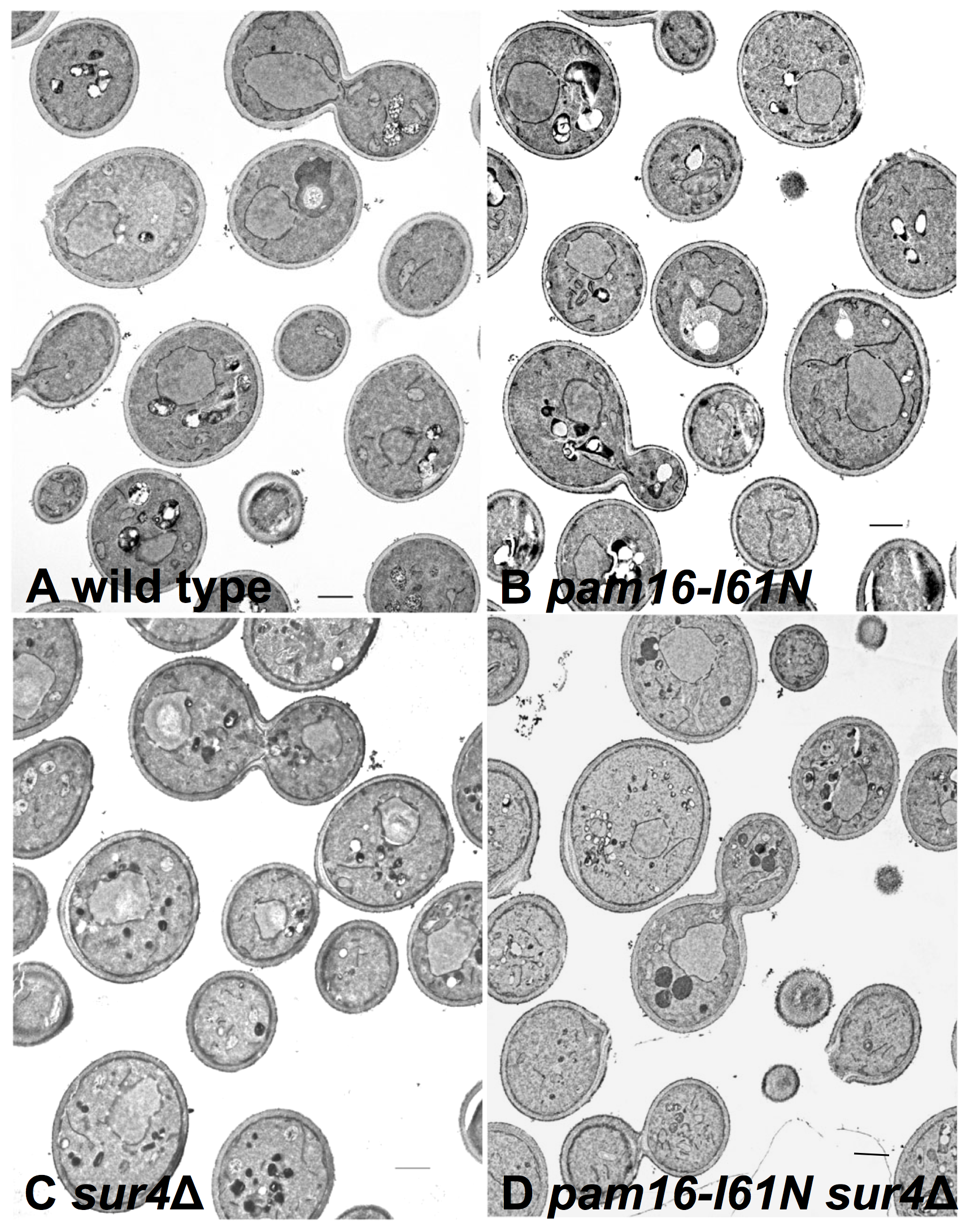

Supplement: Figure S4 — Effects of pam16-I61N and sur4 Δ on yeast morphology. Transmission electron microscopy of yeast strains wt (A), pam16-I61N mutant strain (B), sur4Δ (C), and pam16-I61N sur4Δ (D) are shown in a low power field to demonstrate that the cells depicted in Figure 5 are representative of the entire population. (5,000× magnification; bar = 1 um). (TIF) [file pone.0039428.s004.tif]

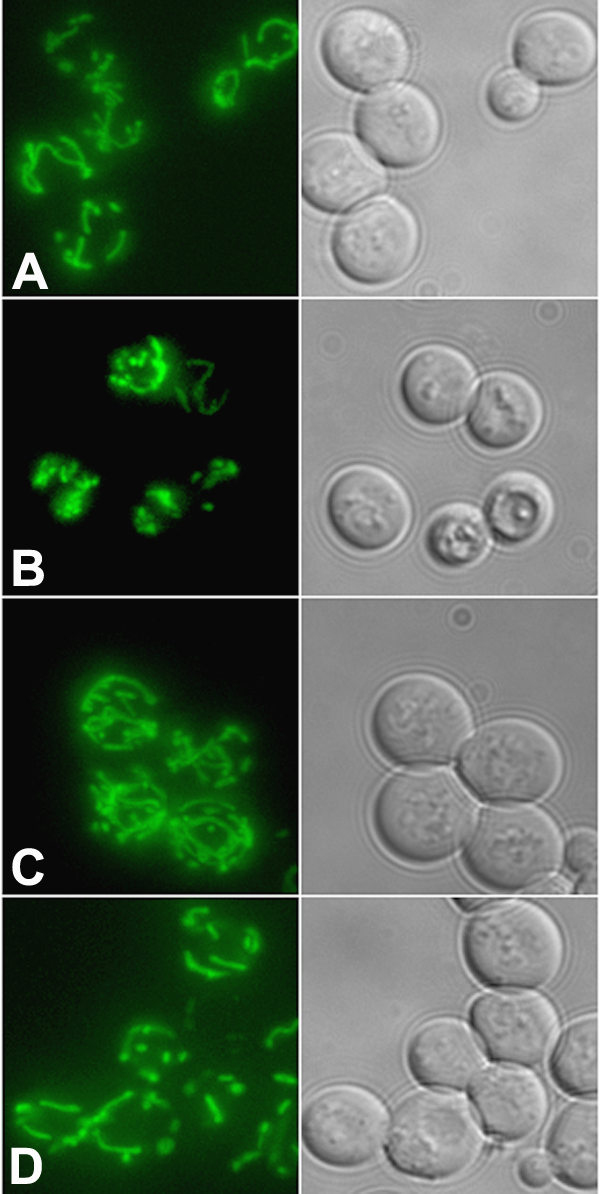

Supplement: Figure S5 — Effects of pam16-I61N and sur4 Δ on mitochondrial morphology. Live-cell fluorescence microscopy of wt (A), pam16-I61N (B), sur4Δ (C) and pam16-I61N sur4Δ (D) containing mitochondrial leader-GFP to display mitochondrial morphology at the semi-permissive temperature (32°C) (left panels) and the corresponding differential interference contrast image are shown in the right panels. The fragmented mitochondria morphology observed in pam16-I61N was restored to the wt elongated reticular mitochondrial morphology in pam16-I61N sur4Δ. (TIF) [file pone.0039428.s005.tif]

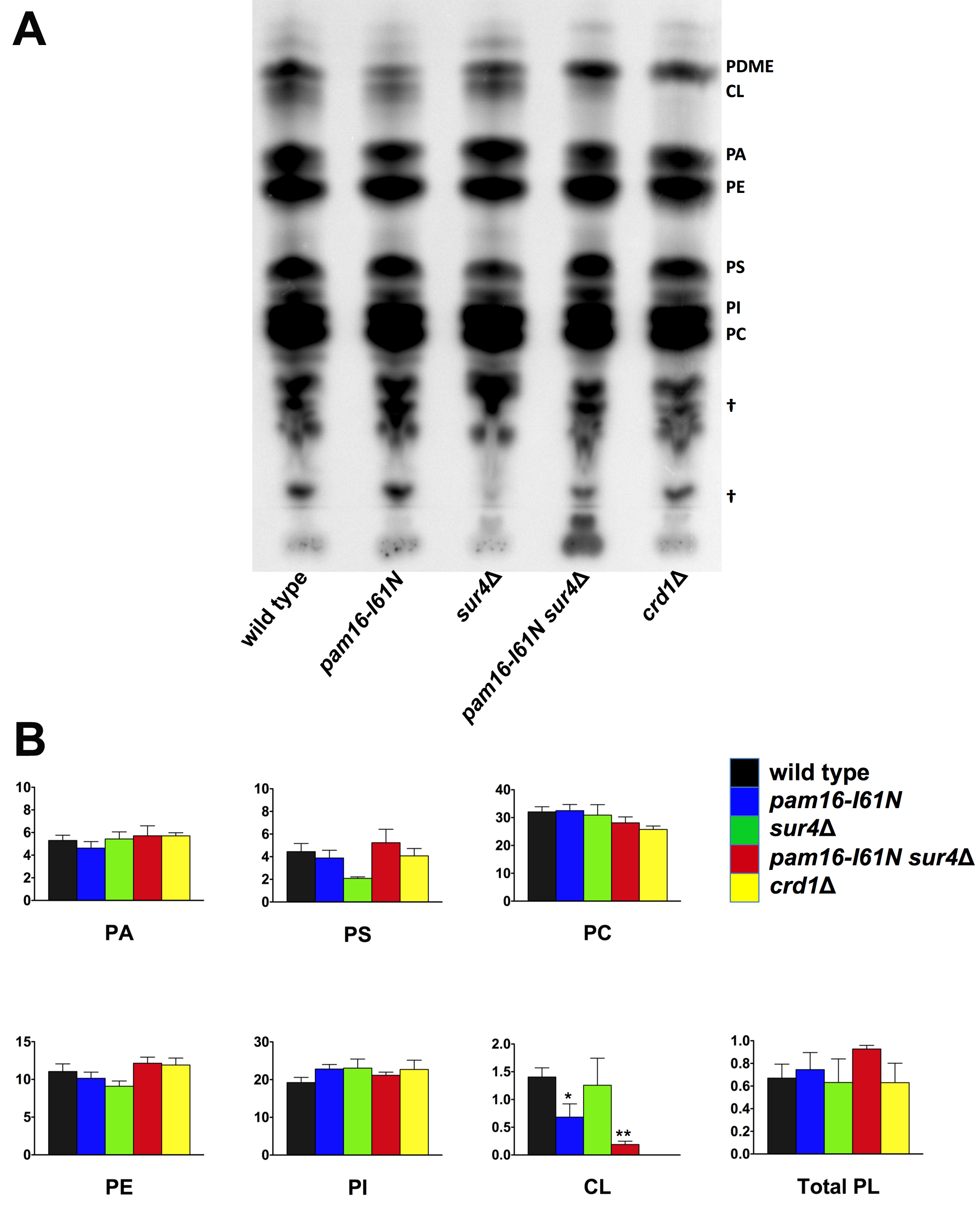

Supplement: Figure S6 — Steady state phospholipid levels in wt, pam16-I61N , sur4 Δ, pam16-I61N sur4 Δ and crd1 Δ. Phospholipids from wt, pam16-I61N, sur4Δ, pam16-I61N sur4Δ and crd1Δ strains were analyzed by thin layer chromatography. (A) Phosphoimage of a representative plate showing the separation of 32P orthophosphate labeled organic extracts from the indicated strains. † Phospholipids at low levels in sur4Δ may be CDP-DAG and LPI/LPC. (B) Quantitation of phospholipids from each strain expressed as % total (cpm specific phospholipid/cpm total phospholipid in sample). Values shown are mean ± SEM. Only cardiolipin levels were significantly different. Wt vs pam16-I61N, *p<0.05; wt vs pam16-I61N sur4Δ, ** p<0.01. Student’s unpaired two-tail t-test. (n = 4). Quantitation of total phospholipid (PL) cpm per lane is shown in the lower right panel. Since wt and pam16-I61N have similar amounts of monovalent phospholipids, these phospholipids were not responsible for the slow growth phenotype and morphological changes occurring in pam16-I61N. pam16-I61N had lower levels of cardiolipin (CL) than wt, and pam16-I61N sur4Δ had even more reduced levels. Therefore CL levels do not account for the suppression of the pam16-I61N fermentative growth defect by deletion of SUR4 but could be involved in the respiratory growth defect (Figure 2). Abbreviations: PDME, phosphatidyldimethylethanolamine; PA, phosphatidic acid; PE, phosphatidylethanolamine; PS, phosphatidylserine; PI, phosphatidylinositol; PC, phosphatidylcholine; LPI/LPC, lysophosphatidylinositol or lysophosphatidic acid; CDP-DAG, cytidine diphosphate diacylglycerol. (TIF) [file pone.0039428.s006.tif]

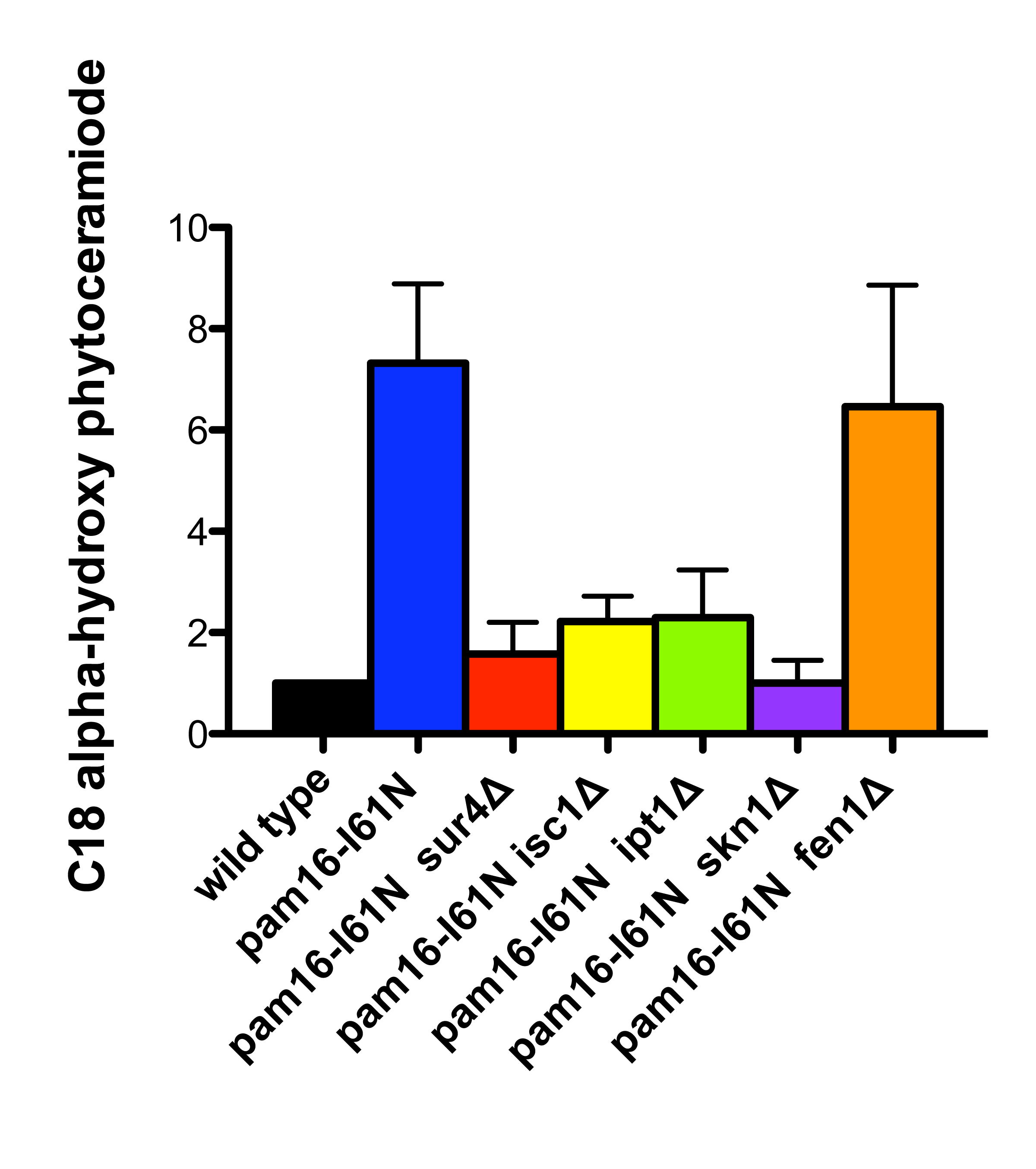

Supplement: Figure S7 — Elevated C18 alpha-hydroxy-phytoceramide levels in pam16-I61N are reduced in pam16-I61N double mutants that suppressed the ts slow growth phenotype. The indicated strains were grown to early log phase in YEPD at 30°C before shifting the temperature to 34°C. Cells were harvested after 6 h at the elevated temperature. The normalized mean level ± SEM of C18 alpha-hydroxy-phytoceramide was determined by mass spectrometry (n = 4). Wt (black), pam16-I61N, (blue), pam16-I61N sur4Δ (red), pam16-I61N isc1Δ (yellow), pam16-I61N ipt1Δ (green), pam16-I61N skn1Δ (purple), pam16-I61N fenΔ (orange). C18 alpha-hydroxy-phytoceramide was 7.5 fold higher in the pam16-I61N strain compared to wt in these experiments. Except for fen1Δ, the weakest suppressor, the double mutant strains had C18 alpha-hydroxy-phytoceramide levels similar to wt. (TIF) [file pone.0039428.s007.tif]
